# Socioeconomic variables and fracture risk in children and adolescents: a population-based study from northern Sweden

**DOI:** 10.1136/bmjopen-2021-053179

**Published:** 2021-10-10

**Authors:** Erik Hedström, Sead Crnalic, Antonia Kullström, Ingeborg Waernbaum

**Affiliations:** 1 Department of Surgical and Perioperative Sciences, Umeå University, Umeå, Sweden; 2 Department of Statistics, Uppsala University, Uppsala, Sweden

**Keywords:** epidemiology, paediatric orthopaedics, community child health

## Abstract

**Objectives:**

Previous studies have investigated the association between socioeconomic characteristics and fractures among children, producing different results. In a population-based study, we previously found an increased risk of fractures among children living in an urban municipality compared with rural municipalities. This study aimed to evaluate the importance of socioeconomic variables for the incidence of fractures among 0–17 year olds.

**Setting, design and outcome measure:**

We present a longitudinal, observational study of a population 0–17 years of age. Data from an injury database were linked with additional socioeconomic data for the population at risk. These were 55 758 individuals residing within the primary catchment area of a regional hospital in northern Sweden. Using the number of fractures as the outcome, we fitted a generalised linear mixed model for a Poisson response with socioeconomic variables at the family level as independent variables while controlling for age, sex and place of residence.

**Results:**

We found a significant association between higher levels of family income and the risk of fracture, rate ratio 1.40 (1.28–1.52) p<0.001 when comparing the highest income quintile to the lowest as well as the number of siblings and the risk of fracture. Children with one or two siblings had a rate ratio of 1.28 (1.19–1.38) p<0.001 when compared with children with no siblings. Parents’ educational level and having a single parent showed no significant association with fractures. The previously observed association between municipalities and fracture risk was less pronounced when taking family-level socioeconomic variables into account.

**Conclusion:**

Our results indicate that children from families with higher income and with siblings are at greater risk of sustaining fractures.

**Strengths and limitations of this study**

- Socioeconomic variables were collected at the individual/family level, the original source being tax records, something that is lacking in many previous studies.
- There were relatively few cases with missing values.
- Using a well-established database and examining the outcome within a well-defined catchment area should ensure that the results are less sensitive to some types of confounding.
- Not all fractures occurring within the catchment area have been registered in the database, minor fractures may not have warranted a visit to the hospital ED, others may have been misdiagnosed, and some cases may have been lost in the registration process.
- Using a different variable for ‘place of residence’, with smaller and more homogenous geographical areas may have yielded other results concerning the variability between areas.

## Background

Fractures are common in children and adolescents. The incidence has consistently been shown to be influenced by age and sex.^1–3^ Previous studies have examined relationships between socioeconomic factors and injuries. Some studies use measurements of socioeconomic status at the individual or family level,^4 5^ while the majority are ecological studies in which geographical units such as parishes or electoral wards are described and compared with respect to socioeconomic variables and injury rates.^6–13^ A few studies have analysed the importance of socioeconomics at both the individual and area levels in multilevel designs.^14 15^

A number of studies have reported an association between measures of social vulnerability or deprivation and injuries. Higher rates of injuries resulting in visits to emergency departments (EDs) have been reported in areas with lower socioeconomic status defined by the Townsend index^6 14^ and in areas with a higher percentage of people living below the poverty line.^7^ Stark and colleagues found a higher rate of fractures in deprived neighbourhoods than in affluent neighbourhoods.^9^ Overpeck and colleagues reported a greater number of medically attended injuries among children in single-parent households.^16^ Other studies have found no significant association^11 13^ or even reverse associations with increased rates of injuries among children from more affluent circumstances.^5 8^

In our previous work, we described the dependences of sex and age on fractures among children and adolescents in Umeå and its surrounding municipalities. We investigated the incidence in different municipalities within the catchment area and found that children living in the four most rural municipalities, namely, Nordmaling, Robertsfors, Bjurholm and Vindeln (NRBV), had significantly fewer fractures than their peers in the more densely populated municipality of Umeå.^17^

**Figure 1:**
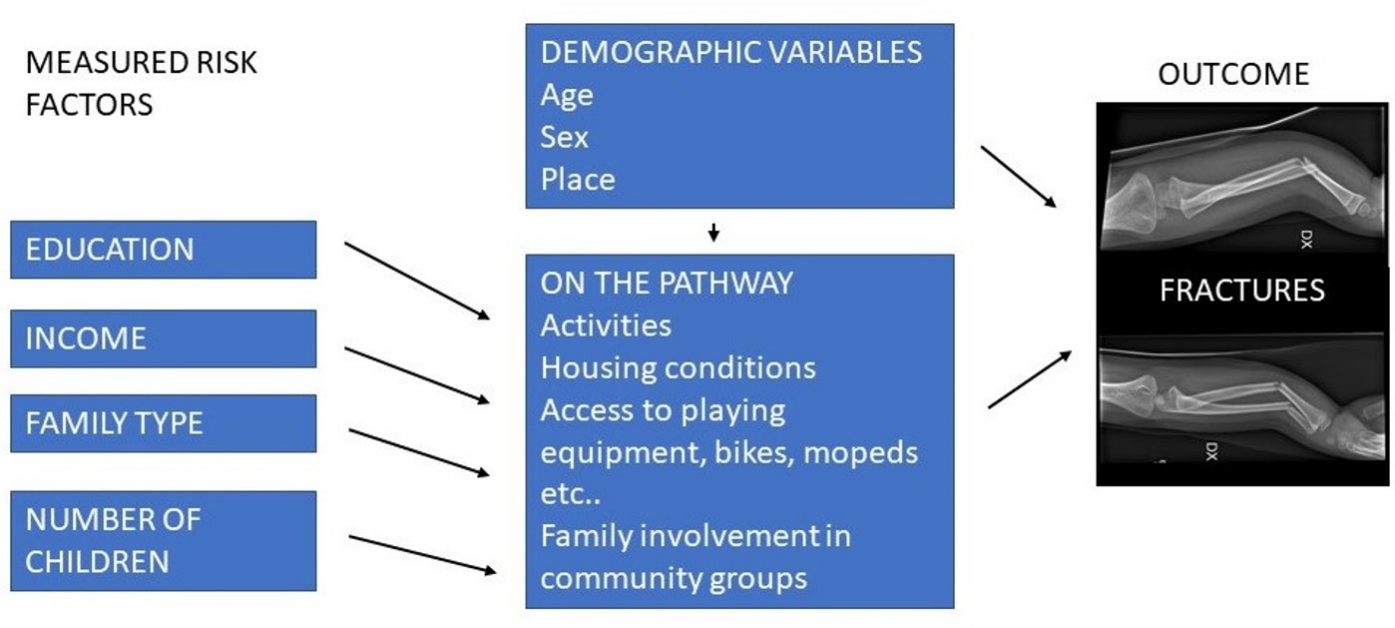
Conceptual model of the relationship between variables and outcome.

This longitudinal, observational study aims to explore the relationships between socioeconomic variables such as parents’ educational level, employment status and income as well as the number of siblings and having a single parent and the risk of fracture in children aged 0–17 years in northern Sweden. The outcome was any fracture leading to a visit to the ED at Umeå University Hospital, which is the sole hospital serving the catchment area. A conceptual model of the relationship between our measured variables and the outcome is illustrated in figure 1.

## Materials and methods

The population at risk was defined as those aged 0–17 years of age living in Umeå and its surrounding municipalities between 1998 and 2010. Because of the longitudinal design of this study, where both explanatory variables and the outcome were measured on a yearly basis, the composition of the population changed over time. Each year, some children were born into, or moved into the population while other children reached adulthood or moved out of the catchment area. This means individuals had varying exposure time, that is, the number of years that they were part of the population at risk. The average population at risk for the time period was 29 817. The characteristics of the municipalities within the catchment area have been described in our previous work.^17^

The Swedish Initiative for Research on Microdata in the Social And Medical Sciences (SIMSAM) is aimed at promoting innovative, interdisciplinary research using microdata. The SIMSAM database includes data from a number of Swedish registries. The fracture data used in this study originally came from the injury database (IDB) at Umeå University Hospital. Socioeconomic data were collected from Statistics Sweden (SCB). The IDB data included information on injuries sustained by patients seeking care at the ED of Umeå University Hospital. Patients with suspected fractures are generally referred to the hospital for diagnosis and treatment. Fractures were in most cases confirmed radiographically.^1^ A small percentage of fractures, such as nose and rib fractures, may have been diagnosed clinically. The quality and validation of IDB data have been described in detail in a previous publication.^1^ For this study, the data included date of fracture, sex, age and a unique personal identifier, which allowed us to link fracture data with the data from SCB. Data from SCB included the same personal identifier and a household identifier, which made it possible to link children and parents/households. For adults, the administrative data contained information on annual employment status, disposable income, highest attained educational level and civil status each year. For these variables, the percentage of missing values ranged between 0.1% and 0.6%. Missing values were excluded from the regression analysis. To control for possible bias due to missing values, we also performed a multiple imputation of the data after our initial analysis. Age, income and number of siblings were originally quantitative, discrete variables but were treated as categorical in the final regression analysis. Income was adjusted for family size and divided into quintiles. Other variables were divided into categories. The outcome for the analysis was the number of fracture events per year.

The project was delayed for reasons beyond our control and this is why the data are somewhat dated at the time of this report.

### Patient and public involvement

No patients were involved in the design, conduct or reporting of this study. Nor have they so far been involved in the dissemination plans of the research. Results will be communicated to the public with help of the communications department at Umeå University.

### Statistical analysis

The outcome, that is, number of fractures/year, is quantitative and possibly zero-inflated; accordingly, a Poisson regression model was first fitted. In addition, to take into account the repeated measurement for individuals, a generalised linear mixed effects model (glmm) was used, incorporating a random effect for the individuals and fixed effects for the demographic and socioeconomic risk factors under study.

For each individual, we used yearly records from linked administrative data from 1998 to 2010. The response variable, number of fractures, and demographic and socioeconomic predictor variables are described in table 1. A zero-inflated generalised linear mixed model for count data was fitted taking into account the dependence structure of the data. All analyses were performed in the R statistical software.^18^ We used the glmmTMB package and the glmmTMB function for the model fit. The CIs for the risk factors were obtained by the delta method using package msm and function deltamethod. Imputation of missing data for a supplementary analysis was performed using the R software, package mice.^18^ Here, the default regression options were used for imputation of the missing values for the variables; number of siblings, family type, parents level of education and family income.

**Table 1:** Distribution of socioeconomic variables

Independent variable		Children with no fracture	Children with fracture
Number of siblings	Mean number (SD)	2.18 (1.10)	2.30 (1.03)
	Missing	24 (<0.01%)	2 (<0.01%)
Family income	Mean income SEK in thousands (SD)	370 (167)	393 (172)
	Median income SEK in thousands	363	379
	Missing	184 (0.4%)	12 (0.2%)
Family type	Cohabiting	40 743 (83.1%)	5715 (84.7%)
	Single parent	8074 (16.5%)	1019 (15.1%)
	Missing	191 (0.4%)	16 (0.2%)
Parents’ level of education	Both parents with primary school education only	1108 (2.3%)	96 (2.2%)
	One or both parents with upper secondary school education but no university level education	18 147 (37.0%)	2810 (41.6%)
	One or both parents w. university level education	28 988 (59.1%)	3826 (58.9%)
	Missing	18 (0.3%)	783 (1.4%)

Percentages within each column and variable.

SEK, Swedish Kronor.

## Results

A total of 55 758 individuals 0–17 years of age were included in the study population. The mean exposure time per individual was 7 years. There were 28 675 boys (51%) and 27 083 girls (49%). One or more fractures were sustained by 6750 individuals (12%) during the study period. In this group of children with fractures, 4006 (59.3%) were boys. Table 1 shows the distribution of values for socioeconomic variables between the group of children with no fracture and fracture. The table also shows the number of missing values per variable.

In the fitted Poisson regression model, there was a significant association between sex, age and fracture incidence. The results are displayed in table 2, including estimates of the regression coefficient β, rate ratios (RRs) with 95% CIs and p values. Boys had an RR of 1.44 (1.36–1.52) in comparison to girls. There was a significant association between family income level and fracture incidence. Those in families with the highest income quintile had an RR of 1.40 (1.28–1.52) in comparison to the lowest quintile families (table 2). Children with siblings had a significantly higher rate of fractures than those who lived in single-child households, RR 1.28 (1.18–1.38) for two–three-children families and RR 1.35 (1.24–1.47) if there were three or more children. There was no significant association between the number of fractures and parents’ educational attainment or single-parent households. There was no statistically significant association between municipality and fractures. Children living in Umeå had an RR of 1.08 (1.00–1.16) compared with children living in most rural municipalities.

**Table 2:** Results of Poisson regression

	Estimated β	RR	95% CI for RR	P value
Intercept	−5.872			
Age 0–5 years	Reference			
6–11 years		1.80	1.68 to 1.92	<0.001
12–17 years		1.89	1.77 to 2.03	<0.001
Sex (boys, girls as reference)	0.361	1.44	1.36 to 1.52	<0.001
Rural municipalities NRBV	Reference			
Umeå	0.0766	1.08	1.00 to 1.16	0.051
Income first quintile	Reference			
Second quintile	0.1043	1.11	1.03 to 1.20	0.009
Third quintile	0.2043	1.23	1.13 to 1.33	<0.001
Fourth quintile	0.2695	1.31	1.21 to 1.42	<0.001
Fifth quintile	0.3361	1.40	1.28 to 1.52	<0.001
Siblings none	Reference			
Siblings (one to two)	0.248	1.28	1.19 to 1.38	<0.001
Siblings (three or more)	0.302	1.35	1.24 to 1.47	<0.001
Single parent (cohabiting as reference)	−0.035	0.97	0.90 to 1.03	0.304
Both parents only primary education	Reference			
No parent with university education	0.200	1.22	0.98 to 1.53	0.081
One parent with university education	0.148	1.16	0.93 to 1.45	0.198

Rate ratios (RR) with 95% CI and p values. For categorical values, the RR is interpreted such that the given RR is relative to the reference category within that variable. NRBV is a grouping of the four most rural municipalities, namely, Nordmaling, Robertsfors, Bjurholm and Vindeln.

Our repeated regression analysis including imputed values did not affect the observed associations or the significance of results Online supplemental table 1 shows the descriptives using imputed values, and in online supplemental table 2, the regression model was refitted with the complete data set after imputation.

10.1136/bmjopen-2021-053179.supp1 Supplementary data

## Discussion

A higher rate of fractures among boys and variations with growth are well described in many previous reports, and our findings support these studies.^2 3 19^

There was a consistent increase in RR with increasing family income. This could be because children from families with more economic resources more often have accessibility to playing equipment such as trampolines, bicycles and skis. As children become older and start participating in organised sports, the cost of membership fees, equipment, etc may also influence the child’s possibilities depending on the family’s economic resources. In a study from the Swedish Research Council for Sport Science, it was reported that children from families with greater economic capital were more likely to participate in sports.^20^ As visits to the hospital ED were free of charge, we do not believe the association between fracture rates and income is explained by lower accessibility to healthcare services among low-income families.

In previous studies, there were conflicting associations between socioeconomic variables and injuries. Several studies have reported a correlation between injuries requiring medical attention, burns, poisonings and pedestrian injuries and measures of deprivation.^4 6 10 21–23^ In an ecological study, Stark *et al* found that children living in deprived areas had a significantly higher fracture rate than those in affluent areas,^9^ and Menon *et al* reported an association between deprivation and fractures in adolescents.^24^ Two other studies from the UK found no association between fractures in general and measures of deprivation.^8 13^ However, Lyons *et al* did report higher rates of sports-related fractures in affluent areas.^8^ Ramaesh *et al* reported that children from more affluent households were more likely to sustain fractures in connection to sports, while children from more deprived families suffered more fractures from road traffic accidents, falls and blows.^12^ These seemingly contradictory findings probably reflect differences in context, age groups, injury types and choice of socioeconomic variables that define each study.

A reason why children with siblings appeared to be at higher risk of fractures could be because these kids interact with their peers and siblings a greater part of the day. It could also be that the types of interactions differ between siblings compared with non-siblings. Reading reported an increased risk of accident attendance in preschool children with an increased number of siblings.^14^ Other than this study, we are not aware of other studies that have reported the influence of the number of siblings on injury outcome.

We found no association between single-parent households and fractures. Overpeck *et al* reported an increased risk of medically attended non-fatal injuries among children from single-parent households.^16^ There are no other studies known to us investigating fracture rates that have used this variable. We found no association between parents’ educational level and fractures. As in our previous study, we found a higher rate of fractures in Umeå than in the four most rural municipalities (NRBV),^17^ but the association was weaker and did not show statistical significance. This is explained by the inclusion of additional explanatory variables at the individual and family level that could have acted as confounders in the previous analysis. This finding does not rule out the importance of place and environment for the outcome. Using different variables at the area level or a different division of areas could have yielded different results. In a study by Eriksson *et al*, the authors investigated the association between social capital and injuries (not only fractures) in Umeå using a multilevel approach and found that some of the variance in child injuries was explained at the neighbourhood level, even if it was less than the proportion of variance explained at the household level.^15^ They also reported an increased incidence of all medically attended injuries in children (0–12 years) in families where no adult had higher (tertiary level) education compared with families where at least one adult had higher education.^15^ They found no association between income levels and injuries. These differences in associations compared with our results could be explained by the type of injuries that were chosen as outcome but may also have been influenced by the difference in age span.

In observational studies, it is not possible to come to conclusions about causal mechanisms that explain observed dependences, so this is a general limit of the study design. In retrospect, it would have been prudent to have used the activity at injury variable, which was part of the original IDB, in this data set. This could have added to our understanding of the associations between income levels, number of children and fractures. A division of fractures according to severity type may have offered further insights into differences between groups. For instance, if the difference in incidence between income groups was largely due to higher rates of high energy, high severity fractures such as spine, pelvic or femoral fractures this would add to the importance of observed differences.

We are also aware that not all fractures are accounted for in the data. Minor fractures may not have been properly diagnosed or may not have warranted a visit to the hospital ED. In our previous research, we found no indication that the distance to the ED significantly influences the rate of fractures.^17^ The number of fractures is also influenced by the completeness of data and accuracy in registration as has been discussed in our previous work.^1^ Our use of socioeconomic variables was limited by our understanding and experience using these variables. We would also like to acknowledge that there are many variables and plausible risk factors that we have no data on. Due to the age of data, our results may not accurately reflect the current rate of fractures in Umeå and its surroundings. Our previous results showed some variation in incidence over time.^1^ If changes have occurred in the population with respect to socioeconomic conditions, health or activities, this may have altered our results with use of more recent data.

A strength of this study is that it is population based within a well-defined catchment area. Socioeconomic variables, based on tax records, were collected at the individual family level and with very few individuals’ missing data, which is rare in previous work. In some previous studies, data were inferred to the individual level from mean values at the area level, such as post codes or parishes. This may reflect a variability that exists at the area level but does not accurately reflect the impact of individual and family characteristics. We believe that our use of several socioeconomic variables to be a strength, as they may reflect different dimensions of socioeconomic status.

## Conclusions

In a population-based study investigating the possible association between socioeconomic variables and fractures, while controlling for age and sex, we found that children from more affluent families in higher income quintiles had higher rates of fractures. Additionally, children with siblings had higher rates of fractures than children without siblings. The previously observed effect of place, with more fractures occurring in an urban municipality than in more rural municipalities, was no longer significant. From these results, it appeared that the association between socioeconomic individual/family level variables and fracture risk was stronger than the association between place of residence and fracture risk. To advance our knowledge and understanding further, future studies should ideally include timely and relevant information on the injury and injury event. Explanatory variables such as age, sex, activities, family characteristics and socioeconomic factors on the individual level need to be included. At the same time, we need to understand and consider the importance of environment and place. The choice of variables is determined by the research questions and will ultimately be limited by the resources available and the skills and expertise of the researchers involved. The formation of multidisciplinary research teams will likely contribute to the quality of future research.

## Supplementary Material

Author's manuscript

## Data Availability

Data may be obtained from a third party and are not publicly available. The data for this study is not available to the public. Data in the SIMSAM lab is stored on secure servers (off grid) in a room which can only be accessed by authorised staff. The ethical approval and the rules concerning use of the SIMSAM database require that data is not moved from the SIMSAM lab. Questions concerning the use of data and applications for use of data can be made to the steering group of the Umeå SIMSAM lab. https://www.umu.se/en/research/infrastructure/umea-simsam-lab/.
