# Supplementary material for: Socioeconomic variables and fracture risk in children and adolescents: a population-based study from northern Sweden
Source: BMJ Open. 2021 Oct 10;11(10):e053179. doi: 10.1136/bmjopen-2021-053179 (PMC8506859; doi:10.1136/bmjopen-2021-053179)
Supplement: Supplementary data [file bmjopen-2021-053179supp001.pdf]

Supplementary Table 1. Distribution of variables in imputed dataset.

Independent variable		Children with no fracture	Children with fracture
**No. of siblings**	Mean number (SD)	2.18 (1.10)	2.30 (1.03)
**Family income**	Mean income SEK* in thousands (SD)	370 (167)	392 (172)
	Median income SEK* in thousands	363	379
**Family type**	Cohabiting	40902 (83.5%)	5731 (84.9%)
	Single parent	8106 (16.5%)	1019 (15.1%)
**Parents´ level of Education**	Both parents with primary school education only	1120 (2.3%)	96 (2.2%)
	One or both parents with upper secondary school education but no university level education	18434 (37.6%)	2816 (41.7%)
	One or both parents w. university level education	29454 (60.1%)	3838 (56.9%)

*Percentages within each column and variable. *SEK (Swedish Kronor)*

Supplementary Table 2. Results of Poisson regression with imputed dataset

	Estim. β	RR	95% CI for RR	P-value
**Intercept**	-5.872			
**Age 0-5 years**	ref.			
**6-11 years**	0.59	1.80	1.68-1.92	<0.001
**12-17 years**	0.64	1.89	1.77-2.02	<0.001
**Sex (boys, girls as ref.)**	0.361	1.44	1.36-1.51	<0.001
**Rural municipalities NRBV**	ref.			
**Umeå**	0.073	1.08	1.00-1.16	0.059
**Income 1st quintile**	ref			
**2nd quintile**	0.107	1.11	1.03-1.20	0.006
**3rd quintile**	0.207	1.23	1.13-1.33	<0.001
**4th quintile**	0.275	1.32	1.22-1.43	<0.001
**5th quintile**	0.344	1.41	1.29-1.54	<0.001
**Siblings none**	ref.			
**Siblings (1-2)**	0.253	1.29	1.19-1.39	<0.001
**Siblings (3 or more)**	0.313	1.37	1.26-1.49	<0.001
**Single parent (cohabiting as ref.)**	-0.035	0.97	0.90-1.03	0.266
**Both parents only primary educ.**	ref.			
**No parent with university educ.**	0.205	1.23	0.98-1.54	0.073
**One parent with university educ.**	0.153	1.17	0.93-1.46	0.182

Rate ratios (RR) with 95% CI and p-values. For categorical values, the RR is interpreted such that the given RR is relative to the reference category within that variable. NRBV is a grouping of the four most rural municipalities, namely, Nordmaling, Robertsfors, Bjurholm and Vindeln.
