# Supplementary material for: Socioeconomic variables and fracture risk in children and adolescents: a population-based study from northern Sweden
Source: BMJ Open. 2021 Oct 10;11(10):e053179. doi: 10.1136/bmjopen-2021-053179 (PMC8506859; doi:10.1136/bmjopen-2021-053179)
Supplement: Author's manuscript [file bmjopen-2021-053179.draft_revisions.pdf]

BMJ Open is committed to open peer review. As part of this commitment we make the peer review history of every article we publish publicly available.

When an article is published we post the peer reviewers' comments and the authors' responses online. We also post the versions of the paper that were used during peer review. These are the versions that the peer review comments apply to.

The versions of the paper that follow are the versions that were submitted during the peer review process. They are not the versions of record or the final published versions. They should not be cited or distributed as the published version of this manuscript.

BMJ Open is an open access journal and the full, final, typeset and author-corrected version of record of the manuscript is available on our site with no access controls, subscription charges or pay-per-view fees (http://bmjopen.bmj.com).

If you have any questions on BMJ Open's open peer review process please email info.bmjopen@bmj.com

BMJ Open

Socioeconomic impact on the incidence of fractures in children and adolescents – a population-based study from northern Sweden

Journal:	*BMJ Open*
Manuscript ID	bmjopen-2021-053179
Article Type:	Original research
Date Submitted by the Author:	08-May-2021

Complete List of Authors:	Hedström, Erik; Umeå Universitet Medicinska fakulteten, Department of Surgical and Perioperative Sciences<br>Crnalic, Sead; Umeå Universitet Medicinska fakulteten, Department of Surgical and Perioperative Sciences<br>Antonia, Kullström; Umeå Universitet Medicinska fakulteten, Department of Surgical and Perioperative Sciences<br>Ingeborg, Waernbaum; Uppsala University, Department of Statistics

Keywords:	EPIDEMIOLOGY, Paediatric orthopaedics < ORTHOPAEDIC & TRAUMA SURGERY, Community child health < PAEDIATRICS

SCHOLARONE™
Manuscripts

I, the Submitting Author has the right to grant and does grant on behalf of all authors of the Work (as defined in the below author licence), an exclusive licence and/or a non-exclusive licence for contributions from authors who are: i) UK Crown employees; ii) where BMJ has agreed a CC-BY licence shall apply, and/or iii) in accordance with the terms applicable for US Federal Government officers or employees acting as part of their official duties; on a worldwide, perpetual, irrevocable, royalty-free basis to BMJ Publishing Group Ltd ("BMJ") its licensees and where the relevant Journal is co-owned by BMJ to the co-owners of the Journal, to publish the Work in this journal and any other BMJ products and to exploit all rights, as set out in our licence.

The Submitting Author accepts and understands that any supply made under these terms is made by BMJ to the Submitting Author unless you are acting as an employee on behalf of your employer or a postgraduate student of an affiliated institution which is paying any applicable article publishing charge ("APC") for Open Access articles. Where the Submitting Author wishes to make the Work available on an Open Access basis (and intends to pay the relevant APC), the terms of reuse of such Open Access shall be governed by a Creative Commons licence – details of these licences and which Creative Commons licence will apply to this Work are set out in our licence referred to above.

Other than as permitted in any relevant BMJ Author's Self Archiving Policies, I confirm this Work has not been accepted for publication elsewhere, is not being considered for publication elsewhere and does not duplicate material already published. I confirm all authors consent to publication of this Work and authorise the granting of this licence.

For peer review only

Socioeconomic impact on the incidence of fractures in children and adolescents – a population-based study from northern Sweden

Hedström Erik Martin MD*, Crnalic Sead MD PhD*, Kullström Antonia MD*, Waernbaum Ingeborg PhD**

* Department of Surgical and Perioperative Sciences, Umeå University, Sweden
** Department of Statistics, Uppsala University, Sweden

Corresponding author: Erik Martin Hedström email: erik.hedstrom@umu.se

The Corresponding author has the right to grant on behalf of all authors and does grant on behalf of all authors, a non-exclusive licence on a worldwide basis to the BMJ Publishing Group Ltd to permit this article (if accepted) to:
i) publish, reproduce, distribute, display and store the contribution,
ii) translate the contribution into other languages, create adaptations, reprints, include within collections and create summaries, extracts and/or, abstracts of the contribution,
iii) create any other derivative work(s) based on the contribution,
iv) to exploit all subsidiary rights in the contribution,
v) the inclusion of electronic links from the contribution to third party material wherever it may be located; and,
vi) licence any third party to do any or all of the above.

Ethics Ethical approval exists under the heading "Injuries in children and adolescents-a population based study". Dnr 2009-1407-31, Regional Ethics Commitee Umeå.

Ethical approval for the use of socioeconomic data in this study was granted within the SIMSAM project under the heading "Childhood diseases and injuries in a social context". Dnr: 2010-157-31, Regional Ethics Commitee Umeå.

For peer review only

Abstract

Objectives Previous studies have investigated the importance of socioeconomics on fractures among children, producing different results. In a population-based study, we previously found an increased risk of fractures among children living in an urban municipality compared to rural municipalities. This study aimed to evaluate the importance of socioeconomic variables for the incidence of fractures among 0–17-year-olds.

Setting, design and outcome measure Data from an injury database were linked with additional socioeconomic data for the population at risk. These were 55,758 individuals residing within the primary catchment area of a regional hospital in northern Sweden. Using the number of fractures as the outcome, we fitted a generalized linear mixed model for a Poisson response with socioeconomic variables at the family level as independent variables while controlling for age, sex, and place of residence.

Results We found a significant association between higher levels of family income and the risk of fracture as well as the number of siblings and the risk of fracture. Parents' educational level and having a single parent showed no significant association with fractures. The previously observed association between municipalities and fracture risk was less pronounced when taking family level socioeconomic variables into account.

Conclusion Our results indicate that children from families with higher income and with siblings are at greater risk of sustaining fractures.

Strengths and limitations

- Socioeconomic variables were collected at the individual/family level, the original source being tax-records, something that is lacking in many previous studies.
- There were relatively few cases with missing values.
- Using a well-established data base, and examining the outcome within a well-defined catchment area should ensure that the results are less sensitive to some types of confounding.
- Not all fractures occurring within the catchment area have been registered in the database, some may have been misdiagnosed and minor fractures may not have warranted a visit to the hospital ED.
- Using a different variable for "place of residence", with smaller and more homogenous geographical areas may have yielded other results concerning the variability between areas.

For peer review only

Background

Fractures are common in children and adolescents. The incidence has consistently been shown to be influenced by age and sex. [1-3] Previous studies have examined relationships between socioeconomic factors and injuries. Some studies use measurements of socioeconomic status at the individual or family level, [4 5] while the majority are ecological studies in which geographical units such as parishes or electoral wards are described and compared with respect to socioeconomic variables and injury rates, [6-13] A few studies have analysed the importance of socioeconomics at both the individual and area levels in multilevel designs. [14 15]

A number of studies have reported an association between measures of social vulnerability or deprivation and injuries. Higher rates of injuries resulting in visits to emergency departments have been reported in areas with lower socioeconomic status defined by the Townsend index [6 14] and in areas with a higher percentage of people living below the poverty line. [7] Stark and colleagues found a higher rate of fractures in deprived neighbourhoods than in affluent neighbourhoods. [9] Overpeck and colleagues reported a greater number of medically attended injuries among children in single-parent households. [16] Other studies have found no significant association [11 13] or even reverse associations with increased rates of injuries among children from more affluent circumstances. [5 8]

In our previous work, we described the dependences of sex and age on fractures among children and adolescents in Umeå and its surrounding municipalities. We investigated the incidence in different municipalities within the catchment area and found that children living in the four most rural municipalities, namely, Nordmaling, Robertsfors, Bjurholm and Vindeln (NRBV), had significantly fewer fractures than their peers in the more densely populated municipality of Umeå. [17]

This study aims to explore the impact and possible relationships between socioeconomic variables such as parents´ educational level, employment status and income as well as the number of siblings and having a single parent and the risk of fracture.

Materials and methods

The population at risk was defined as those aged 0-17 years of age living in Umeå and its surrounding municipalities between 1998-2010. The average population at risk for this time period was 29,817. The characteristics of the municipalities within the catchment area have been described in our previous work. [17]

The Swedish Initiative for Research on Microdata in the Social And Medical Sciences (SIMSAM) is aimed at promoting innovative, interdisciplinary research using microdata. The SIMSAM database includes data from a number of Swedish registries. The fracture data used in this study originally came from the Injury Database (IDB) at Umeå University Hospital. Socioeconomic data were collected from Statistics Sweden (SCB). The IDB data included information on injuries sustained by patients seeking care at the Emergency Department (ED) of Umeå University Hospital. Fractures were in most cases confirmed radiographically. [1] A small percentage of fractures, such as nose and rib fractures, may have been diagnosed clinically. The quality and validation of IDB data has been described in detail in a previous publication. [1] For this study, the data included date of fracture, sex, age, and a unique personal identifier, which allowed us to link fracture data with the data from SCB. Data from SCB included the same personal identifier and a household identifier, which made it possible to link children and parents/households. For adults, the administrative data contained information on annual employment status, disposable income, highest attained educational level and civil status each year. For these variables, the percentage of missing values ranged between 0.1-0.6%. Missing values were excluded from the regression analysis. Income was adjusted for family

For peer review only - http://bmjopen.bmj.com/site/about/guidelines.xhtml

size and divided into quintiles. Other variables were divided into categories. The outcome for the analysis was the number of fracture events per year.

Patient and public involvement No patients were involved in the design, conduct or reporting of this study. Nor have they so far been involved in the dissemination plans of the research. Results will be communicated to the public with help of the communications department at Umeå University.

Statistical analysis The outcome, i.e., number of fractures/year, is quantitative and possibly zero-inflated; accordingly, a Poisson regression model was first fitted. In addition, to take into account the repeated measurement for individuals, a generalized linear mixed effects model (glmm) was used, incorporating a random effect for the individuals and fixed effects for the demographic and socioeconomic risk factors under study.

For each individual, we used yearly records from linked administrative data from 1998-2010. The response variable, number of fractures, and demographic and socioeconomic predictor variables are described in Table 1. A zero-inflated generalized linear mixed model for count data was fitted taking into account the dependence structure of the data. All analyses were performed in the R statistical software. [18] We used the glmmTMB package and the glmmTMB function for the model fit. The confidence intervals (CIs) for the risk factors were obtained by the delta method using package msm and function deltamethod.

Results

A total of 55,758 individuals 0-17 years of age were included in the study population. The mean exposure time per individual was 7 years. There were 28,675 boys (51%) and 27,083 girls (49%). One or more fractures were sustained by 6750 individuals (12%) during the study period. In this group of children with fractures, 4006 (59.3%) were boys. Table 1 shows the distribution of values for socioeconomic variables between the group of children with no fracture and fracture. The table also shows the number of missing values per variable.

Table 1. Distribution of socioeconomic variables.

Independent variable		Children with no fracture	Children with fracture
**No. of siblings**	Mean number (SD)	2.18 (1.10)	2.30 (1.03)
	Missing	24 (<0.01%)	2 (<0.01%)
**Family income**	Mean income SEK in thousands (SD)	370 (167)	393 (172)
	Missing	184 (0.4%)	12 (0.2%)
**Family type**	Cohabiting	40743 (83.1%)	5715 (84.7%)
	Single parent	8074 (16.5%)	9093 (15.1%)
	Missing	191 (0.4%)	16 (0.2%)
**Parents´ level of Education**	Both parents w. primary school education only	1108 (2.3%)	96 (2.2%)
	One or both parents w. upper secondary school education but no university level education	18147 (37.0%)	2810 (41.6%)
	One or both parents w. university level education	28988 (59.1%)	3826 (58.9%)
	Missing	18 (0.3%)	783 (1.4%)

Percentages within each column and variable.

In the fitted Poisson regression model, there was a significant association between sex, age and fracture incidence. The results are displayed in Table 2, including estimates of the regression coefficient β, rate ratios (RRs) with 95% CIs and p-values. Boys had a RR of 1.44 (1.36-1.52) in

For peer review only

comparison to girls. There was a significant association between family income level and fracture incidence. Those in families with the highest income quintile had a RR of 1.40 (1.28-1.52) in comparison to the lowest quintile families (Figure 2). Children with siblings had a significantly higher rate of fractures than those who lived in single child households, RR 1.28 (1.18-1.38) for 2-3 children families and RR 1.35 (1.24-1.47) if there were 3 or more children. There was no significant association between the number of fractures and parents´ educational attainment or single-parent households. There was no statistically significant association between municipality and fractures. Children living in Umeå had a RR of 1.08 (1.00-1.16) compared to children living in the most rural municipalities.

Table 2. Results of Poisson regression

	Estim. β	RR	95% CI for RR	P-value
**Intercept**	-5.872			
**Age 0-5 years**	ref.			
**6-11 years**		1.80	1.68-1.92	<0.001
**12-17 years**		1.89	1.77-2.03	<0.001
**Sex (boys, girls as ref.)**	0.361	1.44	1.36-1.52	<0.001
**Rural municipalities NRBV**	ref.			
**Umeå**	0.0766	1.08	1.00-1.16	0.051
**Income 1st quintile**	ref			
**2nd quintile**	0.1043	1.11	1.03-1.20	0.009
**3rd quintile**	0.2043	1.23	1.13-1.33	<0.001
**4th quintile**	0.2695	1.31	1.21-1.42	<0.001
**5th quintile**	0.3361	1.40	1.28-1.52	<0.001
**Siblings none**	ref.			
**Siblings (1-2)**	0.248	1.28	1.19-1.38	<0.001
**Siblings (3 or more)**	0.302	1.35	1.24-1.47	<0.001
**Single parent (cohabiting as ref.)**	-0.035	0.97	0.90-1.03	0.304
**Both parents only primary educ.**	ref.			
**No parent with university educ.**	0.200	1.22	0.98-1.53	0.081
**One parent with university educ.**	0.148	1.16	0.93-1.45	0.198

Rate ratios (RR) with 95% CI and p-values. For categorical values, the RR is interpreted such that the given RR is relative to the reference category within that variable. NRBV is a grouping of the four most rural municipalities, namely, Nordmaling, Robertsfors, Bjurholm and Vindeln.

Discussion

A higher rate of fractures among boys and variations with growth are well described in many previous reports, and our findings support these studies. [2 3 19]

There was a consistent increase in RR with increasing family income. This could be because children from families with more economic resources more often have accessibility to playing equipment such as trampolines, bicycles, and skis. As children become older and start participating in organized sports, the cost of membership fees, equipment, etc. may also influence the child´s possibilities depending on the family´s economic resources. In a study from the Swedish Research Council for Sport Science, it was reported that children from families with greater economic capital were more likely to participate in sports. [20] As visits to the hospital ED were free of charge, we do not believe the association between fracture rates and income is explained by lower accessibility to health care services among low-income families.

In previous studies, there were conflicting associations between socioeconomic variables and injuries. Several studies have reported a correlation between injuries requiring medical attention,

For peer review only

burns, poisonings and pedestrian injuries and measures of deprivation. [4 6 10 21-23] In an ecological study, Stark et al found that children living in deprived areas had a significantly higher fracture rate than those in affluent areas, [9] and Menon et al reported an association between deprivation and fractures in adolescents. [24] Two other studies from the UK found no association between fractures in general and measures of deprivation. [8 13] However, Lyons et al did report higher rates of sports-related fractures in affluent areas. [8] Ramaesh et al reported that children from more affluent households were more likely to sustain fractures in connection to sports, while children from more deprived families suffered more fractures from road traffic accidents, falls and blows. [12] These seemingly contradictory findings probably reflect differences in context, age groups, injury types, and choice of socioeconomic variables that define each study.

A reason why children with siblings appeared to be at higher risk of fractures could be because these kids interact with their peers and siblings a greater part of the day. It could also be that the types of interactions differ between siblings compared to non-siblings. Reading reported an increased risk of accident attendance in preschool children with an increased number of siblings. [14] Other than this study, we are not aware of other studies that have reported the influence of the number of siblings on injury outcome.

We found no association between single-parent households and fractures. Overpeck et al reported an increased risk of medically attended nonfatal injuries among children from single-parent households. [16] There are no other studies known to us investigating fracture rates that have used this variable. We found no association between parents' educational level and fractures. As in our previous study, we found a higher rate of fractures in Umeå than in the four most rural municipalities (NRBV), [17] but the association was weaker and did not show statistical significance. This is explained by the inclusion of additional explanatory variables at the individual and family level that could have acted as confounders in the previous analysis. This finding does not rule out the importance of place and environment for the outcome. Using different variables at the area level or a different division of areas could have yielded different results. In a study by Eriksson et al., the authors investigated the association between social capital and injuries in Umeå using a multilevel approach and found that some of the variance in child injuries was explained at the neighbourhood level, even if it was less than the proportion of variance explained at the household level. [15] They also reported an increased incidence of all medically attended injuries in children (0-12 years) in families where no adult had higher (tertiary level) education compared to families where at least one adult had higher education. [15] They found no association between income levels and injuries. These differences in associations compared to our results could be explained by the type of injuries that were chosen as outcome, but may also have been influenced by the difference in age span.

In observational studies, it is not possible to come to conclusions about causal mechanisms that explain observed dependences, so this is a general limit of the study design. Inclusion of a "activity at injury" variable in this dataset might have added to our understanding of the associations between income levels, number of children, and fractures. We are also aware that not all fractures may be accounted for in the data. Minor fractures may not have been properly diagnosed or may not have warranted a visit to the hospital ED. In our previous research, we found no indication that the distance to the ED significantly influences the rate of fractures. [17]

A strength of this study is that it is population-based within a well-defined catchment area. Socioeconomic variables, based on tax records, were collected at the individual family level and with very few individuals missing data, which is rare in previous work. In some previous studies, data were inferred to the individual level from mean values at the area level, such as post codes or parishes. This may reflect a variability that exists at the area level but does not accurately reflect the

For peer review only - http://bmjopen.bmj.com/site/about/guidelines.xhtml

impact of individual and family characteristics. We believe our use of several socioeconomic variables to be a strength, as they may reflect different dimensions of socioeconomic status.

Conclusions

In a population-based study investigating the possible association between socioeconomic variables and fractures, while controlling for age and sex, we found that children from more affluent families in higher income quintiles had higher rates of fractures. Additionally, children with siblings had higher rates of fractures than children without siblings. The previously observed effect of place, with more fractures occurring in an urban municipality than in more rural municipalities, was no longer significant. From these results, it appeared that the association between socioeconomic individual/family level variables and fracture risk was stronger than the association between place of residence and fracture risk.

Contributorship statement All authors have made substantial contributions to the research and production of this manuscript, according to ICMJE guidelines, and have approved the submitted manuscript.

Competing interests All authors have completed the Unified Competing Interest form and declare: no support from any organisation for the submitted work; no financial relationships with any organisations that might have an interest in the submitted work in the previous three years; and no other relationships or activities that could appear to have influenced the submitted work.

Funding This study has been funded by Umeå University and Västerbotten county (FS 2.1.6-2408-18 & RV-940639).

Data sharing statement The data analysed in this study is not available to the public. Socioeconomic data originally came from Statistics Sweden (SCB) and injury data came from the Injury Database at Umeå University Hospital. The combined data was stored within the Umeå SIMSAM lab. Our ethical approval and the rules concerning use of the SIMSAM database require that data is not moved from the SIMSAM lab.

References

1. Hedström EM, Svensson O, Bergström U, et al. Epidemiology of fractures in children and adolescents. *Acta orthopaedica* 2010;81(1):148-53. doi: 10.3109/17453671003628780
2. Rennie L, Court-Brown CM, Mok JYQ, et al. The epidemiology of fractures in children. *Injury* 2007;38(8):913-22. doi: 10.1016/j.injury.2007.01.036
3. Bergman E, Lempesis V, Nilsson J-Å, et al. Time trends in pediatric fractures in a Swedish city from 1950 to 2016. *Acta orthopaedica* 2020;91(5):598-604. doi: 10.1080/17453674.2020.1783484
4. Engström K, Diderichsen F, Laflamme L. Socioeconomic differences in injury risks in childhood and adolescence: a nation-wide study of intentional and unintentional injuries in Sweden. *Injury prevention* 2002;8(2):137-42. doi: 10.1136/ip.8.2.137
5. Ni H, Barnes P, Hardy AM. Recreational injury and its relation to socioeconomic status among school aged children in the US. *Injury prevention* 2002;8(1):60-65. doi: 10.1136/ip.8.1.60
6. Laing GJ, Logan S. Patterns of unintentional injury in childhood and their relation to socio-economic factors. *Public health (London)* 1999;113(6):291-94. doi: 10.1016/S0033-3506(99)00182-1
7. Faelker T, Pickett W, Brison RJ. Socioeconomic differences in childhood injury: a population based epidemiologic study in Ontario, Canada. *Injury prevention* 2000;6(3):203-08. doi: 10.1136/ip.6.3.203

For peer review only

8. Lyons RA, Delahunty AM, Heaven M, et al. Incidence of childhood fractures in affluent and deprived areas: population based study. *BMJ* 2000;320(7228):149-49. doi: 10.1136/bmj.320.7228.149
9. Stark AD, Bennet GC, Stone DH, et al. Association between childhood fractures and poverty: population based study. *BMJ* 2002;324(7335):457-57. doi: 10.1136/bmj.324.7335.457
10. Reimers A, Laflamme L. Neighbourhood social and socio-economic composition and injury risks. *Acta Paediatrica, International Journal of Paediatrics* 2005;94(10):1488-94. doi: 10.1080/08035250510038929
11. Reimers A-M, De Leon AP, Laflamme L. The area-based social patterning of injuries among 10 to 19 year olds Changes over time in the Stockholm County. *BMC public health* 2008;8(1):131-31. doi: 10.1186/1471-2458-8-131
12. Ramaesh R, Clement ND, Rennie L, et al. Social deprivation as a risk factor for fractures in childhood. *The bone & joint journal* 2015;97-B(2):240-45. doi: 10.1302/0301-620X.97B2.34057
13. Baker R, Orton E, Tata LJ, et al. Epidemiology of poisonings, fractures and burns among 0-24 year olds in England using linked health and mortality data. *European journal of public health* 2016;26(6):940-46. doi: 10.1093/eurpub/ckw064
14. Reading R, Langford IH, Haynes R, et al. Accidents to preschool children: comparing family and neighbourhood risk factors. *Social science & medicine (1982)* 1999;48(3):321-30. doi: 10.1016/S0277-9536(98)00311-6
15. Eriksson M, Lindgren U, Ivarsson A, et al. The effect of neighbourhood social capital on child injuries: A gender-stratified analysis. *Health & place* 2019;60:102205-05. doi: 10.1016/j.healthplace.2019.102205
16. Overpeck MD, Jones DH, Trumble AC, et al. Socioeconomic and racial/ethnic factors affecting non-fatal medically attended injury rates in US children. *Injury prevention* 1997;3(4):272-76. doi: 10.1136/ip.3.4.272
17. Hedström EM, Waernbaum I. Incidence of fractures among children and adolescents in rural and urban communities - analysis based on 9,965 fracture events. *Injury epidemiology* 2014;1(1):1-5. doi: 10.1186/2197-1714-1-14
18. R: A Language and Environment for Statistical Computing [program]. Vienna, Austria: R Foundation for Statistical Computing, 2020.
19. Landin LA. Fracture patterns in children : analysis of 8,682 fractures with special reference to incidence, etiology and secular changes in a Swedish urban population 1950-1979. Copenhagen: Munksgaard 1983.
20. Idrotten och (o)jämlikheten: i medlemmarnas eller samhällets intresse? In: Dartsch C, Norberg JR, Pihlblad J, eds. Stockholm, Sweden, 2019.
21. Hippisley-Cox J, Groom L, Kendrick D, et al. Cross sectional survey of socioeconomic variations in severity and mechanism of childhood injuries in Trent 1992-7. *BMJ* 2002;324(7346):1132-34. doi: 10.1136/bmj.324.7346.1132
22. Gilbride SJ, Wild C, Wilson DR, et al. Socio-economic status and types of childhood injury in Alberta: A population based study. *BMC pediatrics* 2006;6(1):30-30. doi: 10.1186/1471-2431-6-30
23. Brown CE, Chishti P, Stone DH. Measuring socio-economic inequalities in the presentation of injuries to a paediatric A&E department: the importance of an epidemiological approach. *Public health (London)* 2005;119(8):721-25. doi: 10.1016/j.puhe.2004.10.009
24. Menon MRG, Walker JL, Court-Brown CM. The epidemiology of fractures in adolescents with reference to social deprivation. *Journal of bone and joint surgery British volume* 2008;90(11):1482-86. doi: 10.1302/0301-620X.90B11.21163 [published Online First: British volume]

For peer review only

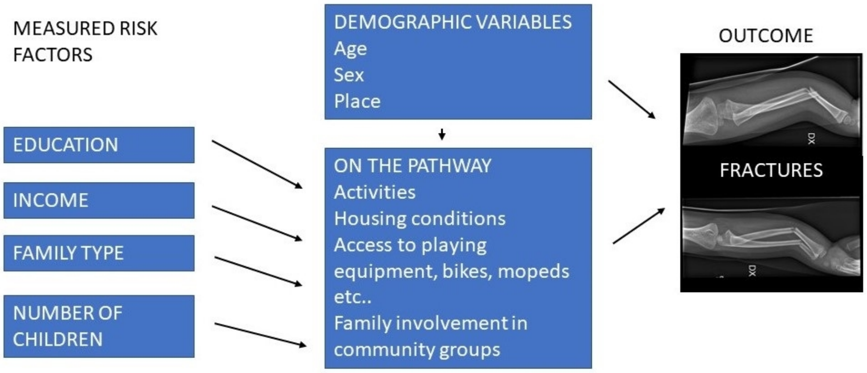

Conceptual model of the relationship between variables and outcome.

291x188mm (96 x 96 DPI)

STROBE Statement—checklist of items that should be included in reports of observational studies

	Item No	Recommendation
**Title and abstract**	1	(*a*) Population-based study page 1 and page 2
		(*b*) Provide in the abstract an informative and balanced summary of what was done and what was found -yes page 2
**Introduction**		
Background/rationale	2	Explain the scientific background and rationale- page 3
Objectives	3	State specific objectives, including any prespecified hypotheses –page 3
**Methods**		
Study design	4	Present key elements of study design early in the paper – page 3 and 4
Setting	5	Population; those 0-17 years of age in principle catchment area of regional hospital in northern Sweden. Period 1998-2010. Page 3
Participants	6	(Observational population-based study. All children and adolescents 0-17 years. Page 3
Variables	7	Clearly define all outcomes, exposures, predictors, potential confounders, and effect modifiers. Give diagnostic criteria, if applicable – yes page 3, figure 1 & table 1
Data sources/ measurement	8*	For each variable of interest, give sources of data and details of methods of assessment (measurement). Describe comparability of assessment methods if there is more than one group. Yes page 3
Bias	9	Describe any efforts to address potential sources of bias – not applicable
Study size	10	Explain how the study size was arrived at – all available data 1998-2010, page 3.
Quantitative variables	11	Explain how quantitative variables were handled in the analyses. If applicable, describe which groupings were chosen and why – yes. page 3 and table 1
Statistical methods	12	(*a*) Describe all statistical methods, including those used to control for confounding – yes page 3 **statistical analysis**
		(*b*) Describe any methods used to examine subgroups and interactions
		(*c*) Explain how missing data were addressed – yes page 3

Continued on next page

For peer review only - http://bmjopen.bmj.com/site/about/guidelines.xhtml

**Results**		
Participants	13*	(a) Report numbers of individuals at each stage of study—eg numbers potentially eligible, examined for eligibility, confirmed eligible, included in the study, completing follow-up, and analysed – not applicable, entire population of age span 0-17, see page 4
		(b) Give reasons for non-participation at each stage -not applicable
		(c) Consider use of a flow diagram – not applicable
Descriptive data	14*	(a) Give characteristics of study participants (eg demographic, clinical, social) and information on exposures and potential confounders – table 1
		(b) Indicate number of participants with missing data for each variable of interest -table 1
Outcome data	15*	*Cohort study*—Report numbers of outcome events or summary measures over time -table 1
		Case-control study—Report numbers in each exposure category, or summary measures of exposure
		Cross-sectional study—Report numbers of outcome events or summary measures
Main results	16	Give unadjusted estimates and, if applicable, confounder-adjusted estimates and their precision (eg, 95% confidence interval). Make clear which confounders were adjusted for and why they were included- Yes, see table 2 and Results page 4 and 5.
Other analyses	17	Report other analyses done—eg analyses of subgroups and interactions, and sensitivity analyses. No subgroups analysed..
**Discussion**		
Key results	18	Summarise key results with reference to study objectives – yes page 5 and 7
Limitations	19	Discuss limitations of the study, taking into account sources of potential bias or imprecision. Discuss both direction and magnitude of any potential bias – yes page 6
Interpretation	20	Give a cautious overall interpretation of results considering objectives, limitations, multiplicity of analyses, results from similar studies, and other relevant evidence -yes page 5 and 6
Generalisability	21	Discuss the generalisability (external validity) of the study results – Some discussion in relation to other results -differences in context and populations. Results are not necessarily applicable on other populations. Page 6.
**Other information**		
Funding	22	Give the source of funding and the role of the funders for the present study and, if applicable, for the original study on which the present article is based. Page 7

*Give information separately for cases and controls in case-control studies and, if applicable, for exposed and unexposed groups in cohort and cross-sectional studies.

Note: An Explanation and Elaboration article discusses each checklist item and gives methodological background and published examples of transparent reporting. The STROBE checklist is best used in conjunction with this article (freely available on the Web sites of PLoS Medicine at http://www.plosmedicine.org/, Annals of Internal Medicine at http://www.annals.org/, and Epidemiology at http://www.epidem.com/). Information on the STROBE Initiative is available at www.strobe-statement.org.

For peer review only

For peer review only - http://bmjopen.bmj.com/site/about/guidelines.xhtml

BMJ Open

Socioeconomic variables and fracture risk in children and adolescents: a population-based study from northern Sweden

Journal:	*BMJ Open*
Manuscript ID	bmjopen-2021-053179.R1
Article Type:	Original research
Date Submitted by the Author:	28-Jul-2021
Complete List of Authors:	Hedström, Erik; Umeå Universitet Medicinska fakulteten, Department of Surgical and Perioperative Sciences<br>Crnalic, Sead; Umeå Universitet Medicinska fakulteten, Department of Surgical and Perioperative Sciences<br>Antonia, Kullström; Umeå Universitet Medicinska fakulteten, Department of Surgical and Perioperative Sciences<br>Ingeborg, Waernbaum; Uppsala University, Department of Statistics
**Primary Subject Heading**:	Epidemiology
Secondary Subject Heading:	Paediatrics
Keywords:	EPIDEMIOLOGY, Paediatric orthopaedics < ORTHOPAEDIC & TRAUMA SURGERY, Community child health < PAEDIATRICS

SCHOLARONE™ Manuscripts

I, the Submitting Author has the right to grant and does grant on behalf of all authors of the Work (as defined in the below author licence), an exclusive licence and/or a non-exclusive licence for contributions from authors who are: i) UK Crown employees; ii) where BMJ has agreed a CC-BY licence shall apply, and/or iii) in accordance with the terms applicable for US Federal Government officers or employees acting as part of their official duties; on a worldwide, perpetual, irrevocable, royalty-free basis to BMJ Publishing Group Ltd ("BMJ") its licensees and where the relevant Journal is co-owned by BMJ to the co-owners of the Journal, to publish the Work in this journal and any other BMJ products and to exploit all rights, as set out in our licence.

The Submitting Author accepts and understands that any supply made under these terms is made by BMJ to the Submitting Author unless you are acting as an employee on behalf of your employer or a postgraduate student of an affiliated institution which is paying any applicable article publishing charge ("APC") for Open Access articles. Where the Submitting Author wishes to make the Work available on an Open Access basis (and intends to pay the relevant APC), the terms of reuse of such Open Access shall be governed by a Creative Commons licence – details of these licences and which Creative Commons licence will apply to this Work are set out in our licence referred to above.

Other than as permitted in any relevant BMJ Author's Self Archiving Policies, I confirm this Work has not been accepted for publication elsewhere, is not being considered for publication elsewhere and does not duplicate material already published. I confirm all authors consent to publication of this Work and authorise the granting of this licence.

For peer review only

Socioeconomic variables and fracture risk in children and adolescents: a population-based study from northern Sweden

Hedström Erik Martin MD*, Crnalic Sead MD PhD*, Kullström Antonia MD*, Waernbaum Ingeborg PhD**

* Department of Surgical and Perioperative Sciences, Umeå University, Sweden
** Department of Statistics, Uppsala University, Sweden

Corresponding author: Erik Martin Hedström email: erik.hedstrom@umu.se

The Corresponding author has the right to grant on behalf of all authors and does grant on behalf of all authors, a non-exclusive licence on a worldwide basis to the BMJ Publishing Group Ltd to permit this article (if accepted) to:
i) publish, reproduce, distribute, display and store the contribution,
ii) translate the contribution into other languages, create adaptations, reprints, include within collections and create summaries, extracts and/or, abstracts of the contribution,
iii) create any other derivative work(s) based on the contribution,
iv) to exploit all subsidiary rights in the contribution,
v) the inclusion of electronic links from the contribution to third party material wherever it may be located; and,
vi) licence any third party to do any or all of the above.

All authors have completed the Unified Competing Interest form and declare: no support from any organisation for the submitted work; no financial relationships with any organisations that might have an interest in the submitted work in the previous three years; and no other relationships or activities that could appear to have influenced the submitted work.

Erik Martin Hedström affirms that the manuscript is an honest, accurate, and transparent account of the study being reported. No important aspects of the study have been omitted.

This study has been funded through public government and regional funds by Umeå University and Region Västerbotten (Västerbotten county).

Ethics Ethical approval exists under the heading "Injuries in children and adolescents-a population-based study". Dnr 2009-1407-31, Regional Ethics Committee Umeå.

Ethical approval for the use of socioeconomic data in this study was granted within the SIMSAM project under the heading "Childhood diseases and injuries in a social context". Dnr: 2010-157-31, Regional Ethics Committee Umeå.

Consent from each participant could not be obtained for this specific study as data was collected in advance of the study.

For peer review only

Abstract

Objectives Previous studies have investigated the association between socioeconomic characteristics and fractures among children, producing different results. In a population-based study, we previously found an increased risk of fractures among children living in an urban municipality compared to rural municipalities. This study aimed to evaluate the importance of socioeconomic variables for the incidence of fractures among 0–17-year-olds.

Setting, design and outcome measure We present a longitudinal, observational study of a population 0-17 years of age. Data from an injury database were linked with additional socioeconomic data for the population at risk. These were 55,758 individuals residing within the primary catchment area of a regional hospital in northern Sweden. Using the number of fractures as the outcome, we fitted a generalized linear mixed model for a Poisson response with socioeconomic variables at the family level as independent variables while controlling for age, sex, and place of residence.

Results We found a significant association between higher levels of family income and the risk of fracture as well as the number of siblings and the risk of fracture. Parents' educational level and having a single parent showed no significant association with fractures. The previously observed association between municipalities and fracture risk was less pronounced when taking family level socioeconomic variables into account.

Conclusion Our results indicate that children from families with higher income and with siblings are at greater risk of sustaining fractures.

Strengths and limitations

- Socioeconomic variables were collected at the individual/family level, the original source being tax-records, something that is lacking in many previous studies.
- There were relatively few cases with missing values.
- Using a well-established data base, and examining the outcome within a well-defined catchment area should ensure that the results are less sensitive to some types of confounding.
- Not all fractures occurring within the catchment area have been registered in the database, some may have been misdiagnosed and minor fractures may not have warranted a visit to the hospital ED. Fractures have been lost in the registration process.
- Using a different variable for "place of residence", with smaller and more homogenous geographical areas may have yielded other results concerning the variability between areas.

For peer review only

Background

Fractures are common in children and adolescents. The incidence has consistently been shown to be influenced by age and sex. [1-3] Previous studies have examined relationships between socioeconomic factors and injuries. Some studies use measurements of socioeconomic status at the individual or family level, [4 5] while the majority are ecological studies in which geographical units such as parishes or electoral wards are described and compared with respect to socioeconomic variables and injury rates, [6-13] A few studies have analysed the importance of socioeconomics at both the individual and area levels in multilevel designs. [14 15]

A number of studies have reported an association between measures of social vulnerability or deprivation and injuries. Higher rates of injuries resulting in visits to emergency departments have been reported in areas with lower socioeconomic status defined by the Townsend index [6 14] and in areas with a higher percentage of people living below the poverty line. [7] Stark and colleagues found a higher rate of fractures in deprived neighbourhoods than in affluent neighbourhoods. [9] Overpeck and colleagues reported a greater number of medically attended injuries among children in single-parent households. [16] Other studies have found no significant association [11 13] or even reverse associations with increased rates of injuries among children from more affluent circumstances. [5 8]

In our previous work, we described the dependences of sex and age on fractures among children and adolescents in Umeå and its surrounding municipalities. We investigated the incidence in different municipalities within the catchment area and found that children living in the four most rural municipalities, namely, Nordmaling, Robertsfors, Bjurholm and Vindeln (NRBV), had significantly fewer fractures than their peers in the more densely populated municipality of Umeå. [17]

This longitudinal, observational study aims to explore the relationships between socioeconomic variables such as parents´ educational level, employment status and income as well as the number of siblings and having a single parent and the risk of fracture in children age 0-17 years in northern Sweden. The outcome was any fracture leading to a visit to the Emergency Department at Umeå University Hospital, which is the sole hospital serving the catchment area. A conceptual model of the relationship between our measured variables and the outcome is illustrated in Figure 1.

Materials and methods

The population at risk was defined as those aged 0-17 years of age living in Umeå and its surrounding municipalities between 1998-2010. Because of the longitudinal design of this study, where both explanatory variables and the outcome were measured on a yearly basis, the composition of the population changed over time. Each year some children were born into, or moved into the population while other children reached adulthood or moved out of the catchment area. This means individuals had varying exposure time, i.e., the number of years that they were part of the population at risk. The average population at risk for the time period was 29,817. The characteristics of the municipalities within the catchment area have been described in our previous work. [17]

The Swedish Initiative for Research on Microdata in the Social And Medical Sciences (SIMSAM) is aimed at promoting innovative, interdisciplinary research using microdata. The SIMSAM database includes data from a number of Swedish registries. The fracture data used in this study originally came from the Injury Database (IDB) at Umeå University Hospital. Socioeconomic data were collected from Statistics Sweden (SCB). The IDB data included information on injuries sustained by patients seeking care at the Emergency Department (ED) of Umeå University Hospital. Patients with suspected fractures are generally referred to the hospital for diagnosis and treatment. Fractures

For peer review only

were in most cases confirmed radiographically. [1] A small percentage of fractures, such as nose and rib fractures, may have been diagnosed clinically. The quality and validation of IDB data has been described in detail in a previous publication. [1] For this study, the data included date of fracture, sex, age, and a unique personal identifier, which allowed us to link fracture data with the data from SCB. Data from SCB included the same personal identifier and a household identifier, which made it possible to link children and parents/households. For adults, the administrative data contained information on annual employment status, disposable income, highest attained educational level and civil status each year. For these variables, the percentage of missing values ranged between 0.1-0.6%. Missing values were excluded from the regression analysis. Age, income and number of siblings were originally quantitative, discrete variables but were treated as categorical in the final regression analysis. Income was adjusted for family size and divided into quintiles. Other variables were divided into categories. The outcome for the analysis was the number of fracture events per year. The project was delayed for reasons beyond our control and this is why the data is somewhat dated at the time of this report.

Patient and public involvement No patients were involved in the design, conduct or reporting of this study. Nor have they so far been involved in the dissemination plans of the research. Results will be communicated to the public with help of the communications department at Umeå University.

Statistical analysis The outcome, i.e., number of fractures/year, is quantitative and possibly zero-inflated; accordingly, a Poisson regression model was first fitted. In addition, to take into account the repeated measurement for individuals, a generalized linear mixed effects model (glmm) was used, incorporating a random effect for the individuals and fixed effects for the demographic and socioeconomic risk factors under study.

For each individual, we used yearly records from linked administrative data from 1998-2010. The response variable, number of fractures, and demographic and socioeconomic predictor variables are described in Table 1. A zero-inflated generalized linear mixed model for count data was fitted taking into account the dependence structure of the data. All analyses were performed in the R statistical software. [18] We used the glmmTMB package and the glmmTMB function for the model fit. The confidence intervals (CIs) for the risk factors were obtained by the delta method using package msm and function deltamethod.

Results

A total of 55,758 individuals 0-17 years of age were included in the study population. The mean exposure time per individual was 7 years. There were 28,675 boys (51%) and 27,083 girls (49%). One or more fractures were sustained by 6750 individuals (12%) during the study period. In this group of children with fractures, 4006 (59.3%) were boys. Table 1 shows the distribution of values for socioeconomic variables between the group of children with no fracture and fracture. The table also shows the number of missing values per variable.

In the fitted Poisson regression model, there was a significant association between sex, age and fracture incidence. The results are displayed in Table 2, including estimates of the regression coefficient β, rate ratios (RRs) with 95% CIs and p-values. Boys had a RR of 1.44 (1.36-1.52) in comparison to girls. There was a significant association between family income level and fracture incidence. Those in families with the highest income quintile had a RR of 1.40 (1.28-1.52) in comparison to the lowest quintile families (Table 2). Children with siblings had a significantly higher rate of fractures than those who lived in single child households, RR 1.28 (1.18-1.38) for 2-3 children families and RR 1.35 (1.24-1.47) if there were 3 or more children. There was no significant

For peer review only

association between the number of fractures and parents´ educational attainment or single-parent households. There was no statistically significant association between municipality and fractures. Children living in Umeå had a RR of 1.08 (1.00-1.16) compared to children living in the most rural municipalities.

Table 1. Distribution of socioeconomic variables.

Independent variable		Children with no fracture	Children with fracture
**No. of siblings**	Mean number (SD)	2.18 (1.10)	2.30 (1.03)
	Missing	24 (<0.01%)	2 (<0.01%)
**Family income**	Mean income SEK* in thousands (SD)	370 (167)	393 (172)
	Median income SEK* in thousands	363	379
	Missing	184 (0.4%)	12 (0.2%)
**Family type**	Cohabiting	40743 (83.1%)	5715 (84.7%)
	Single parent	8074 (16.5%)	9093 (15.1%)
	Missing	191 (0.4%)	16 (0.2%)
**Parents´ level of Education**	Both parents with primary school education only	1108 (2.3%)	96 (2.2%)
	One or both parents with upper secondary school education but no university level education	18147 (37.0%)	2810 (41.6%)
	One or both parents w. university level education	28988 (59.1%)	3826 (58.9%)
	Missing	18 (0.3%)	783 (1.4%)

*Percentages within each column and variable. *SEK (Swedish Kronor)*

Table 2. Results of Poisson regression

	Estim. β	RR	95% CI for RR	P-value
**Intercept**	-5.872			
**Age 0-5 years**	ref.			
**6-11 years**		1.80	1.68-1.92	<0.001
**12-17 years**		1.89	1.77-2.03	<0.001
**Sex (boys, girls as ref.)**	0.361	1.44	1.36-1.52	<0.001
**Rural municipalities NRBV**	ref.			
**Umeå**	0.0766	1.08	1.00-1.16	0.051
**Income 1st quintile**	ref			
**2nd quintile**	0.1043	1.11	1.03-1.20	0.009
**3rd quintile**	0.2043	1.23	1.13-1.33	<0.001
**4th quintile**	0.2695	1.31	1.21-1.42	<0.001
**5th quintile**	0.3361	1.40	1.28-1.52	<0.001
**Siblings none**	ref.			
**Siblings (1-2)**	0.248	1.28	1.19-1.38	<0.001
**Siblings (3 or more)**	0.302	1.35	1.24-1.47	<0.001
**Single parent (cohabiting as ref.)**	-0.035	0.97	0.90-1.03	0.304
**Both parents only primary educ.**	ref.			
**No parent with university educ.**	0.200	1.22	0.98-1.53	0.081
**One parent with university educ.**	0.148	1.16	0.93-1.45	0.198

Rate ratios (RR) with 95% CI and p-values. For categorical values, the RR is interpreted such that the given RR is relative to the reference category within that variable. NRBV is a grouping of the four most rural municipalities, namely, Nordmaling, Robertsfors, Bjurholm and Vindeln.

Discussion

A higher rate of fractures among boys and variations with growth are well described in many previous reports, and our findings support these studies. [2 3 19]

For peer review only

There was a consistent increase in RR with increasing family income. This could be because children from families with more economic resources more often have accessibility to playing equipment such as trampolines, bicycles, and skis. As children become older and start participating in organized sports, the cost of membership fees, equipment, etc. may also influence the child´s possibilities depending on the family´s economic resources. In a study from the Swedish Research Council for Sport Science, it was reported that children from families with greater economic capital were more likely to participate in sports. [20] As visits to the hospital ED were free of charge, we do not believe the association between fracture rates and income is explained by lower accessibility to health care services among low-income families.

In previous studies, there were conflicting associations between socioeconomic variables and injuries. Several studies have reported a correlation between injuries requiring medical attention, burns, poisonings and pedestrian injuries and measures of deprivation. [4 6 10 21-23] In an ecological study, Stark et al found that children living in deprived areas had a significantly higher fracture rate than those in affluent areas, [9] and Menon et al reported an association between deprivation and fractures in adolescents. [24] Two other studies from the UK found no association between fractures in general and measures of deprivation. [8 13] However, Lyons et al did report higher rates of sports-related fractures in affluent areas. [8] Ramaesh et al reported that children from more affluent households were more likely to sustain fractures in connection to sports, while children from more deprived families suffered more fractures from road traffic accidents, falls and blows. [12] These seemingly contradictory findings probably reflect differences in context, age groups, injury types, and choice of socioeconomic variables that define each study.

A reason why children with siblings appeared to be at higher risk of fractures could be because these kids interact with their peers and siblings a greater part of the day. It could also be that the types of interactions differ between siblings compared to non-siblings. Reading reported an increased risk of accident attendance in preschool children with an increased number of siblings. [14] Other than this study, we are not aware of other studies that have reported the influence of the number of siblings on injury outcome.

We found no association between single-parent households and fractures. Overpeck et al reported an increased risk of medically attended nonfatal injuries among children from single-parent households. [16] There are no other studies known to us investigating fracture rates that have used this variable. We found no association between parents' educational level and fractures. As in our previous study, we found a higher rate of fractures in Umeå than in the four most rural municipalities (NRBV), [17] but the association was weaker and did not show statistical significance. This is explained by the inclusion of additional explanatory variables at the individual and family level that could have acted as confounders in the previous analysis. This finding does not rule out the importance of place and environment for the outcome. Using different variables at the area level or a different division of areas could have yielded different results. In a study by Eriksson et al., the authors investigated the association between social capital and injuries in Umeå using a multilevel approach and found that some of the variance in child injuries was explained at the neighbourhood level, even if it was less than the proportion of variance explained at the household level. [15] They also reported an increased incidence of all medically attended injuries in children (0-12 years) in families where no adult had higher (tertiary level) education compared to families where at least one adult had higher education. [15] They found no association between income levels and injuries. These differences in associations compared to our results could be explained by the type of injuries that were chosen as outcome, but may also have been influenced by the difference in age span.

For peer review only

In observational studies, it is not possible to come to conclusions about causal mechanisms that explain observed dependences, so this is a general limit of the study design. In retrospect, it would have been prudent to have used the activity at injury variable, which was part of the original IDB, in this dataset. This could have added to our understanding of the associations between income levels, number of children, and fractures. We are also aware that not all fractures are accounted for in the data. Minor fractures may not have been properly diagnosed or may not have warranted a visit to the hospital ED. In our previous research, we found no indication that the distance to the ED significantly influences the rate of fractures. [17] The number of fractures is also influenced by the completeness of data and accuracy in registration as has been discussed in our previous work[1]. Our use of socioeconomic variables was limited by our understanding and experience using these variables. We would also like to acknowledge that there are many variables and plausible risk factors that we have no data on.

A strength of this study is that it is population-based within a well-defined catchment area. Socioeconomic variables, based on tax records, were collected at the individual family level and with very few individuals missing data, which is rare in previous work. In some previous studies, data were inferred to the individual level from mean values at the area level, such as post codes or parishes. This may reflect a variability that exists at the area level but does not accurately reflect the impact of individual and family characteristics. We believe our use of several socioeconomic variables to be a strength, as they may reflect different dimensions of socioeconomic status.

Conclusions

In a population-based study investigating the possible association between socioeconomic variables and fractures, while controlling for age and sex, we found that children from more affluent families in higher income quintiles had higher rates of fractures. Additionally, children with siblings had higher rates of fractures than children without siblings. The previously observed effect of place, with more fractures occurring in an urban municipality than in more rural municipalities, was no longer significant. From these results, it appeared that the association between socioeconomic individual/family level variables and fracture risk was stronger than the association between place of residence and fracture risk.

Contributors: We hereby confirm that all authors have made substantial contributions to this work. EMH & IW were behind the conception and design of the work and the acquisition of data. EMH, AK, SC, & IW all contributed to the analysis and interpretation of the data. All authors were involved in the drafting and revision of the manuscript. All authors have approved the final version to be published and have agreed to be accountable for all aspects of the work in ensuring that questions related to the accuracy or integrity of any part of the work are appropriately investigated and resolved.

Funding: This study was funded by Region Västerbotten (Västerbotten County) (FS 2.1.6-2408-18 RV-940639) and by Umeå University (FS 2.1.6-2408-18 RV-940639)

Competing Interests: There are no competing interests for any author.

Data sharing statement: The data for this study is not available to the public. Data in the SIMSAM lab is stored on secure servers (off grid) in a room which can only be accessed by authorised staff. The ethical approval and the rules concerning use of the SIMSAM database require that data is not moved from the SIMSAM lab. Questions concerning the use of data and applications for use of data can be made to the steering group of the Umeå SIMSAM lab. https://www.umu.se/en/research/infrastructure/umea-simsam-lab/

For peer review only

References

1. Hedström EM, Svensson O, Bergström U, et al. Epidemiology of fractures in children and adolescents. *Acta orthopaedica* 2010;81(1):148-53. doi: 10.3109/17453671003628780
2. Rennie L, Court-Brown CM, Mok JYQ, et al. The epidemiology of fractures in children. *Injury* 2007;38(8):913-22. doi: 10.1016/j.injury.2007.01.036
3. Bergman E, Lempesis V, Nilsson J-Å, et al. Time trends in pediatric fractures in a Swedish city from 1950 to 2016. *Acta orthopaedica* 2020;91(5):598-604. doi: 10.1080/17453674.2020.1783484
4. Engström K, Diderichsen F, Laflamme L. Socioeconomic differences in injury risks in childhood and adolescence: a nation-wide study of intentional and unintentional injuries in Sweden. *Injury prevention* 2002;8(2):137-42. doi: 10.1136/ip.8.2.137
5. Ni H, Barnes P, Hardy AM. Recreational injury and its relation to socioeconomic status among school aged children in the US. *Injury prevention* 2002;8(1):60-65. doi: 10.1136/ip.8.1.60
6. Laing GJ, Logan S. Patterns of unintentional injury in childhood and their relation to socio-economic factors. *Public health (London)* 1999;113(6):291-94. doi: 10.1016/S0033-3506(99)00182-1
7. Faelker T, Pickett W, Brison RJ. Socioeconomic differences in childhood injury: a population based epidemiologic study in Ontario, Canada. *Injury prevention* 2000;6(3):203-08. doi: 10.1136/ip.6.3.203
8. Lyons RA, Delahunty AM, Heaven M, et al. Incidence of childhood fractures in affluent and deprived areas: population based study. *BMJ* 2000;320(7228):149-49. doi: 10.1136/bmj.320.7228.149
9. Stark AD, Bennet GC, Stone DH, et al. Association between childhood fractures and poverty: population based study. *BMJ* 2002;324(7335):457-57. doi: 10.1136/bmj.324.7335.457
10. Reimers A, Laflamme L. Neighbourhood social and socio-economic composition and injury risks. *Acta Paediatrica, International Journal of Paediatrics* 2005;94(10):1488-94. doi: 10.1080/08035250510038929
11. Reimers A-M, De Leon AP, Laflamme L. The area-based social patterning of injuries among 10 to 19 year olds Changes over time in the Stockholm County. *BMC public health* 2008;8(1):131-31. doi: 10.1186/1471-2458-8-131
12. Ramaesh R, Clement ND, Rennie L, et al. Social deprivation as a risk factor for fractures in childhood. *The bone & joint journal* 2015;97-B(2):240-45. doi: 10.1302/0301-620X.97B2.34057
13. Baker R, Orton E, Tata LJ, et al. Epidemiology of poisonings, fractures and burns among 0-24 year olds in England using linked health and mortality data. *European journal of public health* 2016;26(6):940-46. doi: 10.1093/eurpub/ckw064
14. Reading R, Langford IH, Haynes R, et al. Accidents to preschool children: comparing family and neighbourhood risk factors. *Social science & medicine (1982)* 1999;48(3):321-30. doi: 10.1016/S0277-9536(98)00311-6
15. Eriksson M, Lindgren U, Ivarsson A, et al. The effect of neighbourhood social capital on child injuries: A gender-stratified analysis. *Health & place* 2019;60:102205-05. doi: 10.1016/j.healthplace.2019.102205
16. Overpeck MD, Jones DH, Trumble AC, et al. Socioeconomic and racial/ethnic factors affecting non-fatal medically attended injury rates in US children. *Injury prevention* 1997;3(4):272-76. doi: 10.1136/ip.3.4.272
17. Hedström EM, Waernbaum I. Incidence of fractures among children and adolescents in rural and urban communities - analysis based on 9,965 fracture events. *Injury epidemiology* 2014;1(1):1-5. doi: 10.1186/2197-1714-1-14
18. R: A Language and Environment for Statistical Computing [program]. Vienna, Austria: R Foundation for Statistical Computing, 2020.

For peer review only

19. Landin LA. Fracture patterns in children : analysis of 8,682 fractures with special reference to incidence, etiology and secular changes in a Swedish urban population 1950-1979. Copenhagen: Munksgaard 1983.
20. Idrotten och (o)jämlikheten: i medlemmarnas eller samhällets intresse? In: Dartsch C, Norberg JR, Pihlblad J, eds. Stockholm, Sweden, 2019.
21. Hippisley-Cox J, Groom L, Kendrick D, et al. Cross sectional survey of socioeconomic variations in severity and mechanism of childhood injuries in Trent 1992-7. *BMJ* 2002;324(7346):1132-34. doi: 10.1136/bmj.324.7346.1132
22. Gilbride SJ, Wild C, Wilson DR, et al. Socio-economic status and types of childhood injury in Alberta: A population based study. *BMC pediatrics* 2006;6(1):30-30. doi: 10.1186/1471-2431-6-30
23. Brown CE, Chishti P, Stone DH. Measuring socio-economic inequalities in the presentation of injuries to a paediatric A&E department: the importance of an epidemiological approach. *Public health (London)* 2005;119(8):721-25. doi: 10.1016/j.puhe.2004.10.009
24. Menon MRG, Walker JL, Court-Brown CM. The epidemiology of fractures in adolescents with reference to social deprivation. *Journal of bone and joint surgery British volume* 2008;90(11):1482-86. doi: 10.1302/0301-620X.90B11.21163 [published Online First: British volume]

For peer review only

Figure legend

Figure 1. Conceptual model of the relationship between variables and outcome.

For peer review only

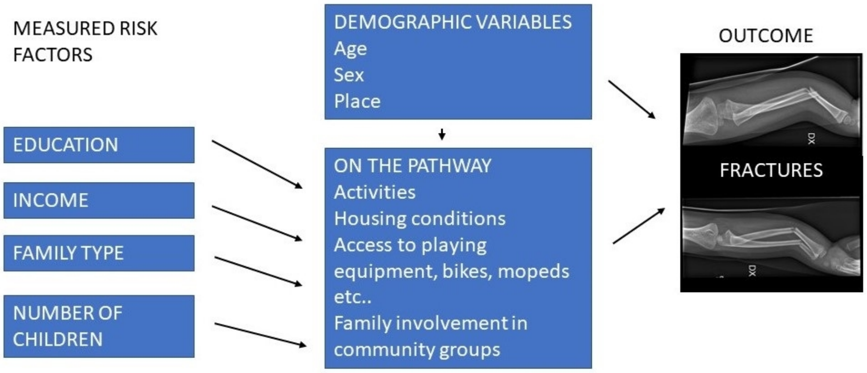

Conceptual model of the relationship between variables and outcome.

291x188mm (96 x 96 DPI)

STROBE Statement—checklist of items that should be included in reports of observational studies

	Item No	Recommendation
**Title and abstract**	1	(*a*) Population-based study page 1 and page 2
		(*b*) Provide in the abstract an informative and balanced summary of what was done and what was found -yes page 2
**Introduction**		
Background/rationale	2	Explain the scientific background and rationale- page 3
Objectives	3	State specific objectives, including any prespecified hypotheses –page 3
**Methods**		
Study design	4	Present key elements of study design early in the paper – page 3 and 4
Setting	5	Population; those 0-17 years of age in principle catchment area of regional hospital in northern Sweden. Period 1998-2010. Page 3
Participants	6	(Observational population-based study. All children and adolescents 0-17 years. Page 3
Variables	7	Clearly define all outcomes, exposures, predictors, potential confounders, and effect modifiers. Give diagnostic criteria, if applicable – yes page 3, figure 1 & table 1
Data sources/ measurement	8*	For each variable of interest, give sources of data and details of methods of assessment (measurement). Describe comparability of assessment methods if there is more than one group. Yes page 3
Bias	9	Describe any efforts to address potential sources of bias – not applicable
Study size	10	Explain how the study size was arrived at – all available data 1998-2010, page 3.
Quantitative variables	11	Explain how quantitative variables were handled in the analyses. If applicable, describe which groupings were chosen and why – yes. page 3 and table 1
Statistical methods	12	(*a*) Describe all statistical methods, including those used to control for confounding – yes page 3 **statistical analysis**
		(*b*) Describe any methods used to examine subgroups and interactions
		(*c*) Explain how missing data were addressed – yes page 3

Continued on next page

For peer review only - http://bmjopen.bmj.com/site/about/guidelines.xhtml

**Results**		
Participants	13*	(a) Report numbers of individuals at each stage of study—eg numbers potentially eligible, examined for eligibility, confirmed eligible, included in the study, completing follow-up, and analysed – not applicable, entire population of age span 0-17, see page 4
		(b) Give reasons for non-participation at each stage -not applicable
		(c) Consider use of a flow diagram – not applicable
Descriptive data	14*	(a) Give characteristics of study participants (eg demographic, clinical, social) and information on exposures and potential confounders – table 1
		(b) Indicate number of participants with missing data for each variable of interest -table 1
Outcome data	15*	*Cohort study*—Report numbers of outcome events or summary measures over time -table 1
		Case-control study—Report numbers in each exposure category, or summary measures of exposure
		Cross-sectional study—Report numbers of outcome events or summary measures
Main results	16	Give unadjusted estimates and, if applicable, confounder-adjusted estimates and their precision (eg, 95% confidence interval). Make clear which confounders were adjusted for and why they were included- Yes, see table 2 and Results page 4 and 5.
Other analyses	17	Report other analyses done—eg analyses of subgroups and interactions, and sensitivity analyses. No subgroups analysed..
**Discussion**		
Key results	18	Summarise key results with reference to study objectives – yes page 5 and 7
Limitations	19	Discuss limitations of the study, taking into account sources of potential bias or imprecision. Discuss both direction and magnitude of any potential bias – yes page 6
Interpretation	20	Give a cautious overall interpretation of results considering objectives, limitations, multiplicity of analyses, results from similar studies, and other relevant evidence -yes page 5 and 6
Generalisability	21	Discuss the generalisability (external validity) of the study results – Some discussion in relation to other results -differences in context and populations. Results are not necessarily applicable on other populations. Page 6.
**Other information**		
Funding	22	Give the source of funding and the role of the funders for the present study and, if applicable, for the original study on which the present article is based. Page 7

*Give information separately for cases and controls in case-control studies and, if applicable, for exposed and unexposed groups in cohort and cross-sectional studies.

Note: An Explanation and Elaboration article discusses each checklist item and gives methodological background and published examples of transparent reporting. The STROBE checklist is best used in conjunction with this article (freely available on the Web sites of PLoS Medicine at http://www.plosmedicine.org/, Annals of Internal Medicine at http://www.annals.org/, and Epidemiology at http://www.epidem.com/). Information on the STROBE Initiative is available at www.strobe-statement.org.

For peer review only - http://bmjopen.bmj.com/site/about/guidelines.xhtml

BMJ Open

Socioeconomic variables and fracture risk in children and adolescents: a population-based study from northern Sweden

Journal:	*BMJ Open*
Manuscript ID	bmjopen-2021-053179.R2
Article Type:	Original research
Date Submitted by the Author:	23-Sep-2021
Complete List of Authors:	Hedström, Erik; Umeå Universitet Medicinska fakulteten, Department of Surgical and Perioperative Sciences<br>Crnalic, Sead; Umeå Universitet Medicinska fakulteten, Department of Surgical and Perioperative Sciences<br>Antonia, Kullström; Umeå Universitet Medicinska fakulteten, Department of Surgical and Perioperative Sciences<br>Ingeborg, Waernbaum; Uppsala University, Department of Statistics
**Primary Subject Heading**:	Epidemiology
Secondary Subject Heading:	Paediatrics
Keywords:	EPIDEMIOLOGY, Paediatric orthopaedics < ORTHOPAEDIC & TRAUMA SURGERY, Community child health < PAEDIATRICS

SCHOLARONE™
Manuscripts

I, the Submitting Author has the right to grant and does grant on behalf of all authors of the Work (as defined in the below author licence), an exclusive licence and/or a non-exclusive licence for contributions from authors who are: i) UK Crown employees; ii) where BMJ has agreed a CC-BY licence shall apply, and/or iii) in accordance with the terms applicable for US Federal Government officers or employees acting as part of their official duties; on a worldwide, perpetual, irrevocable, royalty-free basis to BMJ Publishing Group Ltd ("BMJ") its licensees and where the relevant Journal is co-owned by BMJ to the co-owners of the Journal, to publish the Work in this journal and any other BMJ products and to exploit all rights, as set out in our licence.

The Submitting Author accepts and understands that any supply made under these terms is made by BMJ to the Submitting Author unless you are acting as an employee on behalf of your employer or a postgraduate student of an affiliated institution which is paying any applicable article publishing charge ("APC") for Open Access articles. Where the Submitting Author wishes to make the Work available on an Open Access basis (and intends to pay the relevant APC), the terms of reuse of such Open Access shall be governed by a Creative Commons licence – details of these licences and which Creative Commons licence will apply to this Work are set out in our licence referred to above.

Other than as permitted in any relevant BMJ Author's Self Archiving Policies, I confirm this Work has not been accepted for publication elsewhere, is not being considered for publication elsewhere and does not duplicate material already published. I confirm all authors consent to publication of this Work and authorise the granting of this licence.

For peer review only

Socioeconomic variables and fracture risk in children and adolescents: a population-based study from northern Sweden

Hedström Erik Martin MD*, Crnalic Sead MD PhD*, Kullström Antonia MD*, Waernbaum Ingeborg PhD**

* Department of Surgical and Perioperative Sciences, Umeå University, Sweden
** Department of Statistics, Uppsala University, Sweden

Corresponding author: Erik Martin Hedström   email: erik.hedstrom@umu.se

The Corresponding author has the right to grant on behalf of all authors and does grant on behalf of all authors, a non-exclusive licence on a worldwide basis to the BMJ Publishing Group Ltd to permit this article (if accepted) to:
i) publish, reproduce, distribute, display and store the contribution,
ii) translate the contribution into other languages, create adaptations, reprints, include within collections and create summaries, extracts and/or, abstracts of the contribution,
iii) create any other derivative work(s) based on the contribution,
iv) to exploit all subsidiary rights in the contribution,
v) the inclusion of electronic links from the contribution to third party material wherever it may be located; and,
vi) licence any third party to do any or all of the above.

All authors have completed the Unified Competing Interest form and declare: no support from any organisation for the submitted work; no financial relationships with any organisations that might have an interest in the submitted work in the previous three years; and no other relationships or activities that could appear to have influenced the submitted work.

Erik Martin Hedström affirms that the manuscript is an honest, accurate, and transparent account of the study being reported. No important aspects of the study have been omitted.

This study has been funded through public government and regional funds by Umeå University and Region Västerbotten (Västerbotten county).

Ethics Ethical approval exists under the heading “Injuries in children and adolescents-a population-based study”. Dnr 2009-1407-31, Regional Ethics Committee Umeå.

Ethical approval for the use of socioeconomic data in this study was granted within the SIMSAM project under the heading “Childhood diseases and injuries in a social context”. Dnr: 2010-157-31, Regional Ethics Committee Umeå.

Consent from each participant could not be obtained for this specific study as data was collected in advance of the study.

For peer review only

Abstract

Objectives Previous studies have investigated the association between socioeconomic characteristics and fractures among children, producing different results. In a population-based study, we previously found an increased risk of fractures among children living in an urban municipality compared to rural municipalities. This study aimed to evaluate the importance of socioeconomic variables for the incidence of fractures among 0–17-year-olds.

Setting, design and outcome measure We present a longitudinal, observational study of a population 0-17 years of age. Data from an injury database were linked with additional socioeconomic data for the population at risk. These were 55,758 individuals residing within the primary catchment area of a regional hospital in northern Sweden. Using the number of fractures as the outcome, we fitted a generalized linear mixed model for a Poisson response with socioeconomic variables at the family level as independent variables while controlling for age, sex, and place of residence.

Results We found a significant association between higher levels of family income and the risk of fracture, rate ratio 1.40 (1.28-1.52) $p<0.001$ when comparing the highest income quintile to the lowest, as well as the number of siblings and the risk of fracture. Children with one or two siblings had a rate ratio of 1.28 (1.19-1.38) $p<0.001$ when compared to children with no siblings. Parents' educational level and having a single parent showed no significant association with fractures. The previously observed association between municipalities and fracture risk was less pronounced when taking family level socioeconomic variables into account.

Conclusion Our results indicate that children from families with higher income and with siblings are at greater risk of sustaining fractures.

Strengths and limitations

- Socioeconomic variables were collected at the individual/family level, the original source being tax-records, something that is lacking in many previous studies.
- There were relatively few cases with missing values.
- Using a well-established database, and examining the outcome within a well-defined catchment area should ensure that the results are less sensitive to some types of confounding.
- Not all fractures occurring within the catchment area have been registered in the database, minor fractures may not have warranted a visit to the hospital ED, others may have been misdiagnosed, and some cases may have been lost in the registration process.
- Using a different variable for "place of residence", with smaller and more homogenous geographical areas may have yielded other results concerning the variability between areas.

For peer review only

Background

Fractures are common in children and adolescents. The incidence has consistently been shown to be influenced by age and sex. [1-3] Previous studies have examined relationships between socioeconomic factors and injuries. Some studies use measurements of socioeconomic status at the individual or family level, [4 5] while the majority are ecological studies in which geographical units such as parishes or electoral wards are described and compared with respect to socioeconomic variables and injury rates, [6-13] A few studies have analysed the importance of socioeconomics at both the individual and area levels in multilevel designs. [14 15]

A number of studies have reported an association between measures of social vulnerability or deprivation and injuries. Higher rates of injuries resulting in visits to emergency departments have been reported in areas with lower socioeconomic status defined by the Townsend index [6 14] and in areas with a higher percentage of people living below the poverty line. [7] Stark and colleagues found a higher rate of fractures in deprived neighbourhoods than in affluent neighbourhoods. [9] Overpeck and colleagues reported a greater number of medically attended injuries among children in single-parent households. [16] Other studies have found no significant association [11 13] or even reverse associations with increased rates of injuries among children from more affluent circumstances. [5 8]

In our previous work, we described the dependences of sex and age on fractures among children and adolescents in Umeå and its surrounding municipalities. We investigated the incidence in different municipalities within the catchment area and found that children living in the four most rural municipalities, namely, Nordmaling, Robertsfors, Bjurholm and Vindeln (NRBV), had significantly fewer fractures than their peers in the more densely populated municipality of Umeå. [17]

This longitudinal, observational study aims to explore the relationships between socioeconomic variables such as parents´ educational level, employment status and income as well as the number of siblings and having a single parent and the risk of fracture in children age 0-17 years in northern Sweden. The outcome was any fracture leading to a visit to the Emergency Department at Umeå University Hospital, which is the sole hospital serving the catchment area. A conceptual model of the relationship between our measured variables and the outcome is illustrated in Figure 1.

Materials and methods

The population at risk was defined as those aged 0-17 years of age living in Umeå and its surrounding municipalities between 1998-2010. Because of the longitudinal design of this study, where both explanatory variables and the outcome were measured on a yearly basis, the composition of the population changed over time. Each year some children were born into, or moved into the population while other children reached adulthood or moved out of the catchment area. This means individuals had varying exposure time, i.e., the number of years that they were part of the population at risk. The average population at risk for the time period was 29,817. The characteristics of the municipalities within the catchment area have been described in our previous work. [17]

The Swedish Initiative for Research on Microdata in the Social And Medical Sciences (SIMSAM) is aimed at promoting innovative, interdisciplinary research using microdata. The SIMSAM database includes data from a number of Swedish registries. The fracture data used in this study originally came from the Injury Database (IDB) at Umeå University Hospital. Socioeconomic data were collected from Statistics Sweden (SCB). The IDB data included information on injuries sustained by patients seeking care at the Emergency Department (ED) of Umeå University Hospital. Patients with suspected fractures are generally referred to the hospital for diagnosis and treatment. Fractures

For peer review only

were in most cases confirmed radiographically. [1] A small percentage of fractures, such as nose and rib fractures, may have been diagnosed clinically. The quality and validation of IDB data has been described in detail in a previous publication. [1] For this study, the data included date of fracture, sex, age, and a unique personal identifier, which allowed us to link fracture data with the data from SCB. Data from SCB included the same personal identifier and a household identifier, which made it possible to link children and parents/households. For adults, the administrative data contained information on annual employment status, disposable income, highest attained educational level and civil status each year. For these variables, the percentage of missing values ranged between 0.1-0.6%. Missing values were excluded from the regression analysis. To control for possible bias due to missing values we also performed a multiple imputation of the data after our initial analysis. Age, income and number of siblings were originally quantitative, discrete variables but were treated as categorical in the final regression analysis. Income was adjusted for family size and divided into quintiles. Other variables were divided into categories. The outcome for the analysis was the number of fracture events per year.

The project was delayed for reasons beyond our control and this is why the data is somewhat dated at the time of this report.

Patient and public involvement No patients were involved in the design, conduct or reporting of this study. Nor have they so far been involved in the dissemination plans of the research. Results will be communicated to the public with help of the communications department at Umeå University.

Statistical analysis The outcome, i.e., number of fractures/year, is quantitative and possibly zero-inflated; accordingly, a Poisson regression model was first fitted. In addition, to take into account the repeated measurement for individuals, a generalized linear mixed effects model (glmm) was used, incorporating a random effect for the individuals and fixed effects for the demographic and socioeconomic risk factors under study.

For each individual, we used yearly records from linked administrative data from 1998-2010. The response variable, number of fractures, and demographic and socioeconomic predictor variables are described in Table 1. A zero-inflated generalized linear mixed model for count data was fitted taking into account the dependence structure of the data. All analyses were performed in the R statistical software. [18] We used the glmmTMB package and the glmmTMB function for the model fit. The confidence intervals (CIs) for the risk factors were obtained by the delta method using package msm and function deltamethod. Imputation of missing data for a supplementary analysis was performed using the R software, package mice.[18] Here the default regression options were used for imputation of the missing values for the variables; number of siblings, family type, parents level of education, and family income.

Results

A total of 55,758 individuals 0-17 years of age were included in the study population. The mean exposure time per individual was 7 years. There were 28,675 boys (51%) and 27,083 girls (49%). One or more fractures were sustained by 6750 individuals (12%) during the study period. In this group of children with fractures, 4006 (59.3%) were boys. Table 1 shows the distribution of values for socioeconomic variables between the group of children with no fracture and fracture. The table also shows the number of missing values per variable.

In the fitted Poisson regression model, there was a significant association between sex, age and fracture incidence. The results are displayed in Table 2, including estimates of the regression coefficient β, rate ratios (RRs) with 95% CIs and p-values. Boys had a RR of 1.44 (1.36-1.52) in

comparison to girls. There was a significant association between family income level and fracture incidence. Those in families with the highest income quintile had a RR of 1.40 (1.28-1.52) in comparison to the lowest quintile families (Table 2). Children with siblings had a significantly higher rate of fractures than those who lived in single child households, RR 1.28 (1.18-1.38) for 2-3 children families and RR 1.35 (1.24-1.47) if there were 3 or more children. There was no significant association between the number of fractures and parents´ educational attainment or single-parent households. There was no statistically significant association between municipality and fractures. Children living in Umeå had a RR of 1.08 (1.00-1.16) compared to children living in the most rural municipalities.

Table 1. Distribution of socioeconomic variables.

Independent variable		Children with no fracture	Children with fracture
**No. of siblings**	Mean number (SD)	2.18 (1.10)	2.30 (1.03)
	Missing	24 (<0.01%)	2 (<0.01%)
**Family income**	Mean income SEK* in thousands (SD)	370 (167)	393 (172)
	Median income SEK* in thousands	363	379
	Missing	184 (0.4%)	12 (0.2%)
**Family type**	Cohabiting	40743 (83.1%)	5715 (84.7%)
	Single parent	8074 (16.5%)	1019 (15.1%)
	Missing	191 (0.4%)	16 (0.2%)
**Parents´ level of Education**	Both parents with primary school education only	1108 (2.3%)	96 (2.2%)
	One or both parents with upper secondary school education but no university level education	18147 (37.0%)	2810 (41.6%)
	One or both parents w. university level education	28988 (59.1%)	3826 (58.9%)
	Missing	18 (0.3%)	783 (1.4%)

*Percentages within each column and variable. *SEK (Swedish Kronor)*

Table 2. Results of Poisson regression

	Estim. β	RR	95% CI for RR	P-value
**Intercept**	-5.872			
**Age 0-5 years**	ref.			
**6-11 years**		1.80	1.68-1.92	<0.001
**12-17 years**		1.89	1.77-2.03	<0.001
**Sex (boys, girls as ref.)**	0.361	1.44	1.36-1.52	<0.001
**Rural municipalities NRBV**	ref.			
**Umeå**	0.0766	1.08	1.00-1.16	0.051
**Income 1st quintile**	ref			
**2nd quintile**	0.1043	1.11	1.03-1.20	0.009
**3rd quintile**	0.2043	1.23	1.13-1.33	<0.001
**4th quintile**	0.2695	1.31	1.21-1.42	<0.001
**5th quintile**	0.3361	1.40	1.28-1.52	<0.001
**Siblings none**	ref.			
**Siblings (1-2)**	0.248	1.28	1.19-1.38	<0.001
**Siblings (3 or more)**	0.302	1.35	1.24-1.47	<0.001
**Single parent (cohabiting as ref.)**	-0.035	0.97	0.90-1.03	0.304
**Both parents only primary educ.**	ref.			
**No parent with university educ.**	0.200	1.22	0.98-1.53	0.081
**One parent with university educ.**	0.148	1.16	0.93-1.45	0.198

Rate ratios (RR) with 95% CI and p-values. For categorical values, the RR is interpreted such that the given RR is relative to the reference category within that variable. NRBV is a grouping of the four most rural municipalities, namely, Nordmaling, Robertsfors, Bjurholm and Vindeln.

For peer review only

Our repeated regression analysis including imputed values did not affect the observed associations or the significance of results. Supplementary Table 1 shows the descriptives using imputed values and in supplementary Table 2 the regression model was refitted with the complete data set after imputation.

Discussion

A higher rate of fractures among boys and variations with growth are well described in many previous reports, and our findings support these studies. [2 3 19]

There was a consistent increase in RR with increasing family income. This could be because children from families with more economic resources more often have accessibility to playing equipment such as trampolines, bicycles, and skis. As children become older and start participating in organized sports, the cost of membership fees, equipment, etc. may also influence the child´s possibilities depending on the family´s economic resources. In a study from the Swedish Research Council for Sport Science, it was reported that children from families with greater economic capital were more likely to participate in sports. [20] As visits to the hospital ED were free of charge, we do not believe the association between fracture rates and income is explained by lower accessibility to health care services among low-income families.

In previous studies, there were conflicting associations between socioeconomic variables and injuries. Several studies have reported a correlation between injuries requiring medical attention, burns, poisonings and pedestrian injuries and measures of deprivation. [4 6 10 21-23] In an ecological study, Stark et al found that children living in deprived areas had a significantly higher fracture rate than those in affluent areas, [9] and Menon et al reported an association between deprivation and fractures in adolescents. [24] Two other studies from the UK found no association between fractures in general and measures of deprivation. [8 13] However, Lyons et al did report higher rates of sports-related fractures in affluent areas. [8] Ramaesh et al reported that children from more affluent households were more likely to sustain fractures in connection to sports, while children from more deprived families suffered more fractures from road traffic accidents, falls and blows. [12] These seemingly contradictory findings probably reflect differences in context, age groups, injury types, and choice of socioeconomic variables that define each study.

A reason why children with siblings appeared to be at higher risk of fractures could be because these kids interact with their peers and siblings a greater part of the day. It could also be that the types of interactions differ between siblings compared to non-siblings. Reading reported an increased risk of accident attendance in preschool children with an increased number of siblings. [14] Other than this study, we are not aware of other studies that have reported the influence of the number of siblings on injury outcome.

We found no association between single-parent households and fractures. Overpeck et al reported an increased risk of medically attended nonfatal injuries among children from single-parent households. [16] There are no other studies known to us investigating fracture rates that have used this variable. We found no association between parents' educational level and fractures. As in our previous study, we found a higher rate of fractures in Umeå than in the four most rural municipalities (NRBV), [17] but the association was weaker and did not show statistical significance. This is explained by the inclusion of additional explanatory variables at the individual and family level that could have acted as confounders in the previous analysis. This finding does not rule out the importance of place and environment for the outcome. Using different variables at the area level or a different division of areas could have yielded different results. In a study by Eriksson et al., the

For peer review only

authors investigated the association between social capital and injuries (not only fractures) in Umeå using a multilevel approach and found that some of the variance in child injuries was explained at the neighbourhood level, even if it was less than the proportion of variance explained at the household level. [15] They also reported an increased incidence of all medically attended injuries in children (0-12 years) in families where no adult had higher (tertiary level) education compared to families where at least one adult had higher education. [15] They found no association between income levels and injuries. These differences in associations compared to our results could be explained by the type of injuries that were chosen as outcome, but may also have been influenced by the difference in age span.

In observational studies, it is not possible to come to conclusions about causal mechanisms that explain observed dependences, so this is a general limit of the study design. In retrospect, it would have been prudent to have used the activity at injury variable, which was part of the original IDB, in this dataset. This could have added to our understanding of the associations between income levels, number of children, and fractures. A division of fractures according to severity type may have offered further insights into differences between groups. For instance, if the difference in incidence between income groups was largely due to higher rates of high energy, high severity fractures such as spine, pelvic or femoral fractures this would add to the importance of observed differences.

We are also aware that not all fractures are accounted for in the data. Minor fractures may not have been properly diagnosed or may not have warranted a visit to the hospital ED. In our previous research, we found no indication that the distance to the ED significantly influences the rate of fractures. [17] The number of fractures is also influenced by the completeness of data and accuracy in registration as has been discussed in our previous work[1]. Our use of socioeconomic variables was limited by our understanding and experience using these variables. We would also like to acknowledge that there are many variables and plausible risk factors that we have no data on. Due to the age of data, our results may not accurately reflect the current rate of fractures in Umeå and its surroundings. Our previous results showed some variation in incidence over time.[1] If changes have occurred in the population with respect to socioeconomic conditions, health or activities this may have altered our results with use of more recent data.

A strength of this study is that it is population-based within a well-defined catchment area. Socioeconomic variables, based on tax records, were collected at the individual family level and with very few individuals missing data, which is rare in previous work. In some previous studies, data were inferred to the individual level from mean values at the area level, such as post codes or parishes. This may reflect a variability that exists at the area level but does not accurately reflect the impact of individual and family characteristics. We believe our use of several socioeconomic variables to be a strength, as they may reflect different dimensions of socioeconomic status.

Conclusions

In a population-based study investigating the possible association between socioeconomic variables and fractures, while controlling for age and sex, we found that children from more affluent families in higher income quintiles had higher rates of fractures. Additionally, children with siblings had higher rates of fractures than children without siblings. The previously observed effect of place, with more fractures occurring in an urban municipality than in more rural municipalities, was no longer significant. From these results, it appeared that the association between socioeconomic individual/family level variables and fracture risk was stronger than the association between place of residence and fracture risk. To advance our knowledge and understanding further, future studies should ideally include timely and relevant information on the injury and injury event. Explanatory

For peer review only

variables such as age, sex, activities, family characteristics and socioeconomic factors on the individual level, need to be included. At the same time, we need to understand and consider the importance of environment and place. The choice of variables is determined by the research questions and will ultimately be limited by the resources available and the skills and expertise of the researchers involved. The formation of multidisciplinary research teams will likely contribute to the quality of future research.

Contributors: We hereby confirm that all authors have made substantial contributions to this work. EMH & IW were behind the conception and design of the work and the acquisition of data. EMH, AK, SC, & IW all contributed to the analysis and interpretation of the data. All authors were involved in the drafting and revision of the manuscript. All authors have approved the final version to be published and have agreed to be accountable for all aspects of the work in ensuring that questions related to the accuracy or integrity of any part of the work are appropriately investigated and resolved.

Funding: This study was funded by Region Västerbotten (Västerbotten County) (FS 2.1.6-2408-18 RV-940639) and by Umeå University (FS 2.1.6-2408-18 RV-940639)

Competing Interests: There are no competing interests for any author.

Data sharing statement: The data for this study is not available to the public. Data in the SIMSAM lab is stored on secure servers (off grid) in a room which can only be accessed by authorised staff. The ethical approval and the rules concerning use of the SIMSAM database require that data is not moved from the SIMSAM lab. Questions concerning the use of data and applications for use of data can be made to the steering group of the Umeå SIMSAM lab. https://www.umu.se/en/research/infrastructure/umea-simsam-lab/

References

1. Hedström EM, Svensson O, Bergström U, et al. Epidemiology of fractures in children and adolescents. *Acta orthopaedica* 2010;81(1):148-53. doi: 10.3109/17453671003628780
2. Rennie L, Court-Brown CM, Mok JYQ, et al. The epidemiology of fractures in children. *Injury* 2007;38(8):913-22. doi: 10.1016/j.injury.2007.01.036
3. Bergman E, Lempesis V, Nilsson J-Å, et al. Time trends in pediatric fractures in a Swedish city from 1950 to 2016. *Acta orthopaedica* 2020;91(5):598-604. doi: 10.1080/17453674.2020.1783484
4. Engström K, Diderichsen F, Laflamme L. Socioeconomic differences in injury risks in childhood and adolescence: a nation-wide study of intentional and unintentional injuries in Sweden. *Injury prevention* 2002;8(2):137-42. doi: 10.1136/ip.8.2.137
5. Ni H, Barnes P, Hardy AM. Recreational injury and its relation to socioeconomic status among school aged children in the US. *Injury prevention* 2002;8(1):60-65. doi: 10.1136/ip.8.1.60
6. Laing GJ, Logan S. Patterns of unintentional injury in childhood and their relation to socio-economic factors. *Public health (London)* 1999;113(6):291-94. doi: 10.1016/S0033-3506(99)00182-1
7. Faelker T, Pickett W, Brison RJ. Socioeconomic differences in childhood injury: a population based epidemiologic study in Ontario, Canada. *Injury prevention* 2000;6(3):203-08. doi: 10.1136/ip.6.3.203
8. Lyons RA, Delahunty AM, Heaven M, et al. Incidence of childhood fractures in affluent and deprived areas: population based study. *BMJ* 2000;320(7228):149-49. doi: 10.1136/bmj.320.7228.149

For peer review only

9. Stark AD, Bennet GC, Stone DH, et al. Association between childhood fractures and poverty: population based study. *BMJ* 2002;324(7335):457-57. doi: 10.1136/bmj.324.7335.457
10. Reimers A, Laflamme L. Neighbourhood social and socio-economic composition and injury risks. *Acta Paediatrica, International Journal of Paediatrics* 2005;94(10):1488-94. doi: 10.1080/08035250510038929
11. Reimers A-M, De Leon AP, Laflamme L. The area-based social patterning of injuries among 10 to 19 year olds Changes over time in the Stockholm County. *BMC public health* 2008;8(1):131-31. doi: 10.1186/1471-2458-8-131
12. Ramaesh R, Clement ND, Rennie L, et al. Social deprivation as a risk factor for fractures in childhood. *The bone & joint journal* 2015;97-B(2):240-45. doi: 10.1302/0301-620X.97B2.34057
13. Baker R, Orton E, Tata LJ, et al. Epidemiology of poisonings, fractures and burns among 0-24 year olds in England using linked health and mortality data. *European journal of public health* 2016;26(6):940-46. doi: 10.1093/eurpub/ckw064
14. Reading R, Langford IH, Haynes R, et al. Accidents to preschool children: comparing family and neighbourhood risk factors. *Social science & medicine (1982)* 1999;48(3):321-30. doi: 10.1016/S0277-9536(98)00311-6
15. Eriksson M, Lindgren U, Ivarsson A, et al. The effect of neighbourhood social capital on child injuries: A gender-stratified analysis. *Health & place* 2019;60:102205-05. doi: 10.1016/j.healthplace.2019.102205
16. Overpeck MD, Jones DH, Trumble AC, et al. Socioeconomic and racial/ethnic factors affecting non-fatal medically attended injury rates in US children. *Injury prevention* 1997;3(4):272-76. doi: 10.1136/ip.3.4.272
17. Hedström EM, Waernbaum I. Incidence of fractures among children and adolescents in rural and urban communities - analysis based on 9,965 fracture events. *Injury epidemiology* 2014;1(1):1-5. doi: 10.1186/2197-1714-1-14
18. R: A Language and Environment for Statistical Computing [program]. Vienna, Austria: R Foundation for Statistical Computing, 2020.
19. Landin LA. Fracture patterns in children : analysis of 8,682 fractures with special reference to incidence, etiology and secular changes in a Swedish urban population 1950-1979. Copenhagen: Munksgaard 1983.
20. Idrotten och (o)jämlikheten: i medlemmarnas eller samhällets intresse? In: Dartsch C, Norberg JR, Pihlblad J, eds. Stockholm, Sweden, 2019.
21. Hippisley-Cox J, Groom L, Kendrick D, et al. Cross sectional survey of socioeconomic variations in severity and mechanism of childhood injuries in Trent 1992-7. *BMJ* 2002;324(7346):1132-34. doi: 10.1136/bmj.324.7346.1132
22. Gilbride SJ, Wild C, Wilson DR, et al. Socio-economic status and types of childhood injury in Alberta: A population based study. *BMC pediatrics* 2006;6(1):30-30. doi: 10.1186/1471-2431-6-30
23. Brown CE, Chishti P, Stone DH. Measuring socio-economic inequalities in the presentation of injuries to a paediatric A&E department: the importance of an epidemiological approach. *Public health (London)* 2005;119(8):721-25. doi: 10.1016/j.puhe.2004.10.009
24. Menon MRG, Walker JL, Court-Brown CM. The epidemiology of fractures in adolescents with reference to social deprivation. *Journal of bone and joint surgery British volume* 2008;90(11):1482-86. doi: 10.1302/0301-620X.90B11.21163 [published Online First: British volume]

For peer review only

Figure legend

Figure 1. Conceptual model of the relationship between variables and outcome.

For peer review only

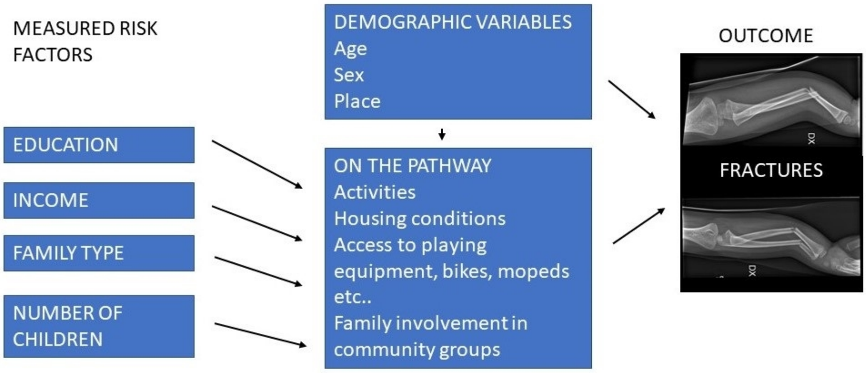

Conceptual model of the relationship between variables and outcome.

291x188mm (96 x 96 DPI)

Supplementary Table 1. Distribution of variables in imputed dataset.

Independent variable		Children with no fracture	Children with fracture
**No. of siblings**	Mean number (SD)	2.18 (1.10)	2.30 (1.03)
**Family income**	Mean income SEK* in thousands (SD)	370 (167)	392 (172)
	Median income SEK* in thousands	363	379
**Family type**	Cohabiting	40902 (83.5%)	5731 (84.9%)
	Single parent	8106 (16.5%)	1019 (15.1%)
**Parents´ level of Education**	Both parents with primary school education only	1120 (2.3%)	96 (2.2%)
	One or both parents with upper secondary school education but no university level education	18434 (37.6%)	2816 (41.7%)
	One or both parents w. university level education	29454 (60.1%)	3838 (56.9%)

*Percentages within each column and variable. *SEK (Swedish Kronor)*

Supplementary Table 2. Results of Poisson regression with imputed dataset

	Estim. β	RR	95% CI for RR	P-value
**Intercept**	-5.872			
**Age 0-5 years**	ref.			
**6-11 years**	0.59	1.80	1.68-1.92	<0.001
**12-17 years**	0.64	1.89	1.77-2.02	<0.001
**Sex (boys, girls as ref.)**	0.361	1.44	1.36-1.51	<0.001
**Rural municipalities NRBV**	ref.			
**Umeå**	0.073	1.08	1.00-1.16	0.059
**Income 1st quintile**	ref			
**2nd quintile**	0.107	1.11	1.03-1.20	0.006
**3rd quintile**	0.207	1.23	1.13-1.33	<0.001
**4th quintile**	0.275	1.32	1.22-1.43	<0.001
**5th quintile**	0.344	1.41	1.29-1.54	<0.001
**Siblings none**	ref.			
**Siblings (1-2)**	0.253	1.29	1.19-1.39	<0.001
**Siblings (3 or more)**	0.313	1.37	1.26-1.49	<0.001
**Single parent (cohabiting as ref.)**	-0.035	0.97	0.90-1.03	0.266
**Both parents only primary educ.**	ref.			
**No parent with university educ.**	0.205	1.23	0.98-1.54	0.073
**One parent with university educ.**	0.153	1.17	0.93-1.46	0.182

Rate ratios (RR) with 95% CI and p-values. For categorical values, the RR is interpreted such that the given RR is relative to the reference category within that variable. NRBV is a grouping of the four most rural municipalities, namely, Nordmaling, Robertsfors, Bjurholm and Vindeln.

For peer review only

STROBE Statement—checklist of items that should be included in reports of observational studies

	Item No	Recommendation
**Title and abstract**	1	(*a*) Population-based study page 1 and page 2
		(*b*) Provide in the abstract an informative and balanced summary of what was done and what was found -yes page 2
**Introduction**		
Background/rationale	2	Explain the scientific background and rationale- page 3
Objectives	3	State specific objectives, including any prespecified hypotheses –page 3
**Methods**		
Study design	4	Present key elements of study design early in the paper – page 3 and 4
Setting	5	Population; those 0-17 years of age in principle catchment area of regional hospital in northern Sweden. Period 1998-2010. Page 3
Participants	6	(Observational population-based study. All children and adolescents 0-17 years. Page 3
Variables	7	Clearly define all outcomes, exposures, predictors, potential confounders, and effect modifiers. Give diagnostic criteria, if applicable – yes page 3, figure 1 & table 1
Data sources/ measurement	8*	For each variable of interest, give sources of data and details of methods of assessment (measurement). Describe comparability of assessment methods if there is more than one group. Yes page 3
Bias	9	Describe any efforts to address potential sources of bias – not applicable
Study size	10	Explain how the study size was arrived at – all available data 1998-2010, page 3.
Quantitative variables	11	Explain how quantitative variables were handled in the analyses. If applicable, describe which groupings were chosen and why – yes. page 3 and table 1
Statistical methods	12	(*a*) Describe all statistical methods, including those used to control for confounding – yes page 3 **statistical analysis**
		(*b*) Describe any methods used to examine subgroups and interactions
		(*c*) Explain how missing data were addressed – yes page 3

Continued on next page

For peer review only

For peer review only - http://bmjopen.bmj.com/site/about/guidelines.xhtml

**Results**		
Participants	13*	(a) Report numbers of individuals at each stage of study—eg numbers potentially eligible, examined for eligibility, confirmed eligible, included in the study, completing follow-up, and analysed – not applicable, entire population of age span 0-17, see page 4
		(b) Give reasons for non-participation at each stage -not applicable
		(c) Consider use of a flow diagram – not applicable
Descriptive data	14*	(a) Give characteristics of study participants (eg demographic, clinical, social) and information on exposures and potential confounders – table 1
		(b) Indicate number of participants with missing data for each variable of interest -table 1
Outcome data	15*	*Cohort study*—Report numbers of outcome events or summary measures over time -table 1
		Case-control study—Report numbers in each exposure category, or summary measures of exposure
		Cross-sectional study—Report numbers of outcome events or summary measures
Main results	16	Give unadjusted estimates and, if applicable, confounder-adjusted estimates and their precision (eg, 95% confidence interval). Make clear which confounders were adjusted for and why they were included- Yes, see table 2 and Results page 4 and 5.
Other analyses	17	Report other analyses done—eg analyses of subgroups and interactions, and sensitivity analyses. No subgroups analysed..
**Discussion**		
Key results	18	Summarise key results with reference to study objectives – yes page 5 and 7
Limitations	19	Discuss limitations of the study, taking into account sources of potential bias or imprecision. Discuss both direction and magnitude of any potential bias – yes page 6
Interpretation	20	Give a cautious overall interpretation of results considering objectives, limitations, multiplicity of analyses, results from similar studies, and other relevant evidence -yes page 5 and 6
Generalisability	21	Discuss the generalisability (external validity) of the study results – Some discussion in relation to other results -differences in context and populations. Results are not necessarily applicable on other populations. Page 6.
**Other information**		
Funding	22	Give the source of funding and the role of the funders for the present study and, if applicable, for the original study on which the present article is based. Page 7

*Give information separately for cases and controls in case-control studies and, if applicable, for exposed and unexposed groups in cohort and cross-sectional studies.

Note: An Explanation and Elaboration article discusses each checklist item and gives methodological background and published examples of transparent reporting. The STROBE checklist is best used in conjunction with this article (freely available on the Web sites of PLoS Medicine at http://www.plosmedicine.org/, Annals of Internal Medicine at http://www.annals.org/, and Epidemiology at http://www.epidem.com/). Information on the STROBE Initiative is available at www.strobe-statement.org.

For peer review only
